# Delayed Degradation and Impaired Dendritic Delivery of Intron-Lacking *EGFP*-*Arc*/*Arg3.1* mRNA in *EGFP-Arc* Transgenic Mice

**DOI:** 10.3389/fnmol.2017.00435

**Published:** 2018-01-31

**Authors:** Oswald Steward, Kelly Matsudaira Yee, Shannon Farris, Patricia S. Pirbhoy, Paul Worley, Kohji Okamura, Hiroyuki Okuno, Haruhiko Bito

**Affiliations:** ^1^Reeve-Irvine Research Center, University of California, Irvine, Irvine, CA, United States; ^2^Department of Anatomy and Neurobiology, University of California, Irvine, Irvine, CA, United States; ^3^Department of Neurobiology and Behavior, University of California, Irvine, Irvine, CA, United States; ^4^Center for the Neurobiology of Learning and Memory, University of California, Irvine, Irvine, CA, United States; ^5^Johns Hopkins School of Medicine, Baltimore, MD, United States; ^6^Department of Systems BioMedicine, National Research Institute for Child Health and Development, Tokyo, Japan; ^7^Medical Innovation Center, Kyoto University Graduate School of Medicine, Kyoto, Japan; ^8^Department of Neurochemistry, University of Tokyo Graduate School of Medicine, Tokyo, Japan

**Keywords:** LTP, synaptic plasticity, protein synthesis, dendrite, dendritic mRNA, dendritic spines, immediate early gene, nonsense-mediated decay

## Abstract

*Arc* is a unique immediate early gene (IEG) whose expression is induced as synapses are modified during learning. Newly-synthesized *Arc* mRNA is rapidly transported throughout dendrites and localizes near recently activated synapses. *Arc* mRNA levels are regulated by rapid degradation, which is accelerated by synaptic activity in a translation-dependent process. One possible mechanism is nonsense-mediated mRNA decay (NMD), which depends on the presence of a splice junction in the 3′UTR. Here, we test this hypothesis using transgenic mice that express *EGFP-Arc*. Because the transgene was constructed from *Arc* cDNA, it lacks intron structures in the 3′UTR that are present in the endogenous *Arc* gene. NMD depends on the presence of proteins of the exon junction complex (EJC) downstream of a stop codon, so *EGFP-Arc mRNA* should not undergo NMD. Assessment of *Arc* mRNA rundown in the presence of the transcription inhibitor actinomycin-D confirmed delayed degradation of *EGFP-Arc* mRNA. *EGFP-Arc* mRNA and protein are expressed at much higher levels in transgenic mice under basal and activated conditions but *EGFP-Arc* mRNA does not enter dendrites efficiently. In a physiological assay in which cycloheximide (CHX) was infused after induction of *Arc* by seizures, there were increases in endogenous *Arc* mRNA levels consistent with translation-dependent *Arc* mRNA decay but this was not seen with *EGFP-Arc* mRNA. Taken together, our results indicate: (1) *Arc* mRNA degradation occurs via a mechanism with characteristics of NMD; (2) rapid dendritic delivery of newly synthesized *Arc* mRNA after induction may depend in part on prior splicing of the 3′UTR.

## Significance

Studies of the immediate early gene *Arc* have revealed fundamental cell biological mechanisms of mRNA transport into dendrites and localization at active synapses and mechanisms of *Arc* mRNA transcription and degradation. Here, we show that two key aspects of *Arc* mRNA dynamics are dependent on introns in the 3′UTR of *Arc* mRNA. In transgenic mice expressing an *EGFP-Arc* transgene with the *Arc-EGFP* coding regions and *Arc* 3′UTR that lacks introns, degradation of *EGFP-Arc* mRNA is delayed and dendritic transport is impaired in comparison to endogenous *Arc* mRNA. Our findings elucidate features of *Arc* mRNA turnover and dendritic transport and highlight the usefulness of *EGFP-Arc* transgenic mice for studies of the functional significance of Arc expression dynamics and dendritic transport.

## Introduction

The immediate early gene *Arc* (Lyford et al., 1995), also known as *Arg 3.1* (Link et al., 1995), has been implicated in synaptic modifications that underlie memory storage (Steward et al., 2014). *Arc* transcription is induced by learning experiences or strong synaptic activity, and *Arc* is unique amongst IEGs in that newly-synthesized *Arc* mRNA is rapidly transported throughout dendrites (Link et al., 1995; Lyford et al., 1995). If synapses are strongly activated as *Arc* mRNA moves into dendrites, *Arc* mRNA localizes selectively near the active synapses (Steward et al., 1998). Patterns of synaptic activation that induce *Arc* transcription simultaneously trigger *Arc* mRNA degradation (Farris et al., 2014). Indeed, it is a combination of selective docking of newly-synthesized *Arc* mRNA near active synapses and activity-dependent *Arc* mRNA degradation that sharpens the selectivity of *Arc* localization in activated dendritic domains (Farris et al., 2014).

An important, but poorly-understood aspect of *Arc* is its expression dynamics. Rapid activity-dependent induction is consistent with Arc's putative role in synaptic plasticity. Arc protein is subject to ubiquitination and proteosomal degradation (Mabb et al., 2014), but mechanisms underlying rapid degradation of *Arc* mRNA are not worked out. A possible mechanism for *Arc* mRNA decay is nonsense-mediated mRNA decay (NMD), which is triggered when a translating ribosome reaches the stop codon and interacts with the exon junction complex (EJC) at a downstream splice site (Giorgi et al., 2007). The *Arc* gene consists of a single open-reading frame exon and two introns in its 3′UTR. After pre-mRNA processing, EJC proteins remain bound to *Arc* mRNA as it moves into the cytoplasm, and the presence of a splice site with EJC's more than about 70 nt downstream of a stop codon is the canonical signal for NMD. It is not clear, however, whether activity-driven degradation of *Arc* mRNA occurs via this mechanism.

A pivotal test of the hypothesis is to determine if *Arc* transcripts that were never spliced (and thus do not have associated EJC proteins) are degraded in the same way as endogenous *Arc* mRNA. To test this, the present study uses a line of transgenic mice in which an *EGFP-Arc* fusion gene that includes the 3′ and 5′UTRs of *Arc* is expressed under the control of the 7kb *Arc* promoter, which recapitulates the activity-dependent expression of *Arc* (Kawashima et al., 2009; Okuno et al., 2012). Importantly, the transgene was created from *Arc* cDNA, which lacks any introns, thus, the transcript is not spliced.

If *Arc* mRNA is degraded by NMD, then: (1) Endogenous *Arc* mRNA should be degraded more quickly than *EGFP-Arc* mRNA; (2) Inhibition of protein synthesis should lead to increases in endogenous *Arc* mRNA (Farris et al., 2014), but not *EGFP-Arc* mRNA levels; and (3) *EGFP-Arc* mRNA should not undergo activity-dependent degradation. Consistent with these predictions, we show here that *EGFP-Arc* mRNA transcription is induced by the same stimuli that induce endogenous *Arc* expression but *EGFP-Arc* mRNA has a longer half-life than endogenous *Arc* mRNA. Unexpectedly, we found that *EGFP-Arc* mRNA was not delivered into dendrites to the same degree as endogenous *Arc* mRNA after induction, suggesting that rapid dendritic delivery of newly synthesized *Arc* mRNA may depend on or be facilitated by prior splicing of the 3′UTR. Although experiments to assess translation-dependence of *Arc* mRNA decay were complicated by the fact that consequences of inhibiting protein synthesis were less striking in mice than previously seen in rats, overall results supported the conclusion that unlike endogenous *Arc* mRNA, *EGFP-Arc* mRNA degradation is not accelerated by mRNA translation. We also found that EGFP-Arc protein also is not degraded with the rapid kinetics of endogenous Arc protein resulting in higher levels of total Arc protein in *EGFP-Arc*-transgenic mice than in wildtype controls.

## Methods

### Experimental animals

Most experiments were carried out using adult male and female mice from our breeding colony that was established using founders derived from frozen embryos of *EGFP-Arc* mice from Okuno and Bito (Okuno et al., 2012). Founder mice were crossed with C57Bl/6 mice to expand the colony and hemizygous mice were then set up in breeding pairs to obtain hemizygous and homozygous transgenic *EGFP-Arc* mice, and controls with only wild type (WT) *Arc*. Some of the neurophysiology experiments used C57Bl/6/J mice obtained from Jackson Labs as detailed in the Results. All procedures involving live animals were approved by the Institutional Animal Care and Use Committees of the University of California Irvine, Kyoto University, and the University of Tokyo.

Mice were genotyped by a commercial vendor (Transnetyx) by TaqMan® qPCR with forward primer: CGTCGTCCTTGAAGAAGATGGT, Reverse Primer: CACATGAAGCAGCACGACTT and an internal oligo with a fluorescent probe (CATGCCCGAAGGCTAC). As noted in the Results, we also found that genotype could be easily determined by *in situ* hybridization.

### Novel enriched environment

To assess *Arc* induction due to a brief learning experience, mice were allowed to explore a novel toy-filled environment for 1 h, which we previously referred to as “unsupervised learning” (Farris et al., 2014). The environment was a 24 × 24 in square plexiglass box with various small items that mice could climb, crawl under, or that moved when touched.

To test whether *Arc* and *EGFP-Arc* mRNA levels depended on ongoing translation as predicted by NMD-dependent mRNA degradation, we tested whether inhibition of protein synthesis with cycloheximide (CHX; 20 mg/kg i.p.) led to increases in mRNA levels as previously reported in rats (Farris et al., 2014). To assess effects of CHX on *Arc* mRNA levels under basal conditions, mice were gently removed from their home cage, received CHX or saline and were returned to their home cage for 1 h. To assess whether CHX led to increased mRNA levels following a learning experience, pairs of mice were allowed to explore the novel environment for 1 h. One mouse in each pair received CHX (20 mg/kg i.p.); the other received saline. Brains from mice that received CHX or saline were mounted together on microscope slides and hybridized or immunostained in the same solutions. This strategy allowed direct comparisons of mRNA and protein levels in brains from mice of the same genotype and with very similar experience with and without CHX.

### Electroconvulsive seizures

A single electroconvulsive seizure (ECS) was induced as described previously (Wallace et al., 1998). Current was passed transcranially (10 mA for 0.5 s) via ear clip electrodes resulting in a generalized tonic/clonic seizure that lasted approximately 15 s.

### Neurophysiology

Mice were anesthetized via intraperitoneal injections of 20% urethane (500 mg/kg body weight) with supplemental doses given approximately every 10 min until the animal was unresponsive to tail pinch. Mice were positioned in a stereotaxic apparatus and burr holes were placed in the skull to allow placement of stimulating and recording electrodes. An insulated monopolar stimulating electrode was positioned stereotaxically at 3.0 mm lateral to the midline at the transverse sinus. The depth of the stimulating electrode was adjusted so as to optimally activate the medial perforant path (MPP) originating from the medial entorhinal cortex (EC)—usually about 3 mm below the cortical surface. Glass recording electrodes filled with 0.9% saline were positioned at 1.5–1.8 mm lateral to the midline, and 2.0 mm posterior to bregma. Electrodes were positioned in the dorsal blade of the dentate gyrus so as to record field potentials from the cell body layer.

### Stimulation paradigm

After positioning the stimulating and recording electrodes, stimulus intensity was set so as to evoke a population spike of 3–6 mV. Single test pulses were delivered at a rate of 1/10 s at the same intensity for 10 min in order to determine baseline response amplitude; measures were the slope of the population EPSP and amplitude of the population spike. Following baseline recordings, 3 rounds of high frequency stimulation was delivered, with each round consisting of ten trains of eight pulses at 400 Hz and each train given at a rate of 1/10 s. After each bout of HFS, a round of ten test pulses was given to determine the extent of LTP. Then, HFS was continued at a rate of one 400 hz train per 10 s for 1 h.

### Assessment of mRNA half-life *in Vivo*

To assess the half-life of *EGFP-Arc* mRNA vs. endogenous *Arc* mRNA *in vivo*, we assessed rundown of mRNA levels after transcriptional block with actinomycin-D (Act-D). *Arc* transcription was induced in transgenic and WT mice by delivering an electroconvulsive seizure (ECS) and time was allowed for mRNA levels to ramp up. Mice received injections of anesthetic (urethane) about 45 min post-ECS and were placed in a stereotaxic device. A recording micropipette containing 2 mg/ml Act-D mannitol in saline was stereotaxically positioned in the dentate gyrus at approximately 1 h post-ECS and a monopolar stimulating electrode was placed in the entorhinal cortex as above. The final position of the Act-D-containing micropipette was adjusted by monitoring evoked responses generated by stimulation of the entorhinal cortex. Micropipettes containing Act-D were left in place for 30 or 60 min (3–4 mice per group at each time point except for 30 min WT where *n* = 2), after which brains were harvested for fluorescent *in situ* hybridization (FISH).

### Tissue preparation

Mice were killed by a lethal injection of the anesthetic Euthasol or Fatal Plus® depending on the IACUC protocol in effect on the date of the experiment. Un-fixed brains were removed and rapidly frozen. For chromogen-based *in situ* hybridization, mice were perfused with 4% paraformaldehyde in phosphate buffered saline (PBS, pH 7.4). Brains were cryoprotected by placing in 25% sucrose overnight and sectioned at 20 μm on a cryostat and slides with sections were stored at −80°C until use.

### *In Situ* hybridization

The cRNA probe for *Arc*/*Arg3.1* was generated as described previously (Steward et al., 1998). The *EGFP* gene from EGFP-N1 was cloned into the BamHI-NotI site of Bluescript KS(+). For transcribing cRNA probes, the Bluescript vector was linearized with BamHI.

Fluorescent *in situ* hybridization (FISH) was performed on 20 μm coronal sections prepared from flash-frozen brains as described by Guzowski et al. (1999). cRNA riboprobes were generated using the Ambion MaxiScript kit and a premixed RNA labeling nucleotide mix containing digoxigenin-labeled UTP (Roche Molecular Biochemicals). Sections were incubated with the digoxigenin-labeled *Arc* antisense riboprobe (1–2 ng/ml) for 16–20 h. Subsequent to washes of various stringencies, slides were incubated with a horseradish peroxidase (HRP)-conjugated antibody to digoxigenin. HRP was detected using the Tyramide Signal Amplification fluorescence (TSA-CY3) kit from Perkin Elmer or from tyramide-FITC synthesized in lab as described (Hopman et al., 1998). In some experiments, sections were also stained with DAPI to mark nuclei. Slides were coverslipped using Vectashield® mounting medium (Vector Laboratories). Comparisons of patterns of labeling and quantitative analyses were done using slides that were hybridized or immunostained in the same run to control for variability in the extent of hybridization/immunostaining.

For chromagen-based *in situ* hybridization slide-mounted sections were post-fixed with 4% paraformaldehyde in 0.1 M PBS for 30 min, then rinsed with 0.5x saline-sodium citrate buffer (0.5xSSC, 0.1%DEPC treated) for 5 min. Sections were treated with Proteinase K (1.25 mg/L) for 30 min, rinsed again with 0.5xSSC (0.1%DEPC treated) for 10 min and air-dried. The sections were covered with 75 μl prehybridization buffer (2xSSC, 25% formamide, 1% Denhardt's reagent, 10% dextran sulfate, 0.5 mg/mL heparin, 0.5 mg/ml yeast tRNA and 0.25 mg/mL of denatured salmon sperm DNA) and incubated at 42°C for 2 h. After the prehybridization, about 0.5 μg of Dig-cRNA probe in 75 μl hybridization buffer was added to each section. The sections were covered with a baked coverslip, and incubated overnight at 55°C in a humidified box with 25% formamide/2xSSC. The next day, the coverslips were removed and sections were washed with 2xSSC/10 mM EDTA twice (10 min each). The sections were treated with RNAse-A for 30 min, and then washed twice with 2xSSC/EDTA (10 min per wash). The stringency wash was 0.5xSSC/10 mMEDTA at 55°C for 2 h. Sections were washed with 0.5xSSC twice (10 min each at room temperature). Alkaline phosphatase conjugated anti-digoxigenin Fab fragment (1:5,000) was used to detect the probes. NBT/BCIP solution was applied overnight at 4°C to detect the alkaline phosphatase. Sections were washed with 100 mM Tris-HCl (pH 8.5)/1 mM EDTA 3 times, 10 min each. Then slides were briefly rinsed with nanopure water twice and covered with Kaiser's glycerol jelly.

#### Quantification of FISH

Quantification of FISH was done using NIH ImageJ. For the experiments involving treatment with CHX vs. saline, images were taken at 20X of an area in the CA1 subfield of the dorsal hippocampus extending from stratum oriens through stratum radiatum (2 different sections, 2 images per section in each animal). All images from a given experiment were taken at the same exposure and imported into a single Tiff file using Adobe Photoshop. The tiff image was opened in ImageJ, and a measuring box was positioned over the CA1 cell body layer. Mean fluorescence intensity over the cell layer of each individual image was assessed using the “Measure” function of NIH ImageJ, and the values were then averaged for each individual animal. Statistical comparisons were done using Prism Version 6 with n = number of animals.

#### Counts of mRNA puncta in dendritic laminae

Counts of mRNA puncta in dendritic laminae were done by imaging sections using confocal microscopy in order to resolve individual fluorescent puncta. Image stacks were collected at 63X from stratum radiatum of area CA1 using a Zeiss LSM700 confocal microscope keeping acquisition parameters constant. The total area of the image was 204.8 μm^2^. Four image stacks taken at an interval of 0.55 μm (total range of 1.65 μm) were collapsed into a maximum intensity image using the Z project function. The image was converted to 8-bit, inverted, and the threshold was adjusted to highlight individual Arc puncta. Particles were counted using NIH ImageJ “particle analysis.”

To assess fluorescence intensity across the cell body and molecular layers, 20X images were acquired at the same exposure and converted to 8-bit gray scale images in ImageJ. Using the ImageJ line function, a region of interest (ROI) line was defined as the length of the cell body layer and the dendritic laminae (250–300 μm in length). This ROI was saved and used for each case and was aligned perpendicular to the cell body layer so that the middle molecular layer was positioned in the middle of the line at 150 μm for 300 μm ROIs and 125 μm for 250 μm ROIs, this ensured that despite differences in the widths of cell body layers, that the region of stimulation (the middle molecular layer) was aligned across cases. Images from chromogen-based *in situ* hybridization acquired in bright field were inverted so that higher density equated to a greater intensity value. Intensities were measured across the line every 10 μm along the length of the 20X image (usually about 10 line measurements per image). Line measurements were averaged across each point in the line to obtain an average value for each point along the line ROI for each case. These average line ROI values were averaged across animals to generate an “average fluorescence intensity (or optical density) vs. distance” graph with average values ± SEM.

### Immunohistochemistry (IHC)

For IHC, slide-mounted sections were fixed in 4% paraformaldehyde, washed, treated with 3% hydrogen peroxide to block endogenous peroxidase and incubated for 1 h in at room temperature in 5% Blocking Reagent from Perkin Elmer (FP1012) before overnight incubation with the primary antibodies. For Arc, we used an antibody from Synaptic Systems (156-003, 1:1,000 dilution). EGFP-Arc fusion protein contains the full length Arc protein, so Arc antibodies detect both endogenous Arc and EGFP-Arc in transgenic mice. To detect EGFP, sections were incubated with an antibody to GFP from Invitrogen (A-11122, 1:1,500).

After incubation in primary antibodies, slides were washed in TBS, incubated for 2 h with HRP-conjugated donkey anti-rabbit IgG secondary antibody (Jackson ImmunoResearch, 711-036-152, 1:500 dilution). HRP was detected using Catalyzed Reporter Deposition (CARD) amplification as described (Hopman et al., 1998). In some cases, sections were stained with Hoechst 33258 (1 μg/ml) to stain nuclei. Slides were then washed with TBS, mounted and coverslipped using Vectashield® (Vector Laboratories).

### SDS-PAGE and western blotting

*EGFP-Arc* tg/tg (*N* = 5) and WT controls from the breeding colony (*N* = 4;10–32 weeks of age) were taken directly from their home cage and euthanized with Fatal Plus (0.5 mL). The left forebrain (cortex and hippocampus) was rapidly dissected on ice and homogenized in 250 μL of Lysis Buffer (50 mM Tris-HCL, pH 7.4, 150 mM NaCl, 5 mM EDTA, 0.05% Triton X-100, 1 mM PMSF, PhosStop phosphatase inhibitor (Roche, cat. 04 906 837 001) and Complete Mini Protease Inhibitor® (Roche, cat. 04 693 124 001). Homogenates were centrifuged at 15,000 × g for 15 min at 4°C. Protein concentrations were determined with the BioRad Protein Assay Kit (cat. 500-0006).

For SDS-PAGE, samples were diluted to 1 μg/ml in 1X Laemmli sample buffer, boiled, and 20 μg of protein were loaded per well and resolved on a 4–20% gradient SDS-PAGE mini-gel (BioRad, cat. 4561095). Proteins were transferred to Immobilon-FL polyvinylidene fluoride membranes (PVDF, cat. IPFL00010) and blocked with Odyssey Blocking buffer (LI-COR, cat. 927-40100 PBS blocking buffer); membranes were incubated with antibodies at a dilution of 1:2,000 rabbit anti-Arc (Synaptic Systems, cat. 156-003) or 1:10,000 mouse anti-β-actin (Sigma, cat. A-5441) followed by incubation in goat anti-rabbit IR dye 800CW (LI-COR, cat. 926-32211) or goat anti-mouse 680CW (LI-COR, cat. 926-68070) at a dilution of 1:15,000. The blots were scanned using the LI-COR system and imaged using Odyssey Fc imagining system. Quantification of fluorescent signal band intensity was done using LI-COR image studio ver.3.1. Values were normalized to the beta-actin loading control.

### Quantitative PCR to determine transgene copy number in the mouse genome

Genomic DNA was extracted from Proteinase K-digested liver lysates of wildtype, hemizygous, or homozygous *EGFP-Arc* Tg mice. The genomic DNA was then purified through Phenol/Chloroform extraction and ethanol precipitation, and analyzed by SYBR green-based qPCR (SYBR Premix Ex Taq II, Takara Bio, Japan) to determine copy numbers of *Arc* ORF and internal control *gapdh* genes. The primers used are follows:

For *Arc*ORF:
Forward, 5′-AGCTGGACCATATGACCACCGG-3′
Reverse, 5′-CAGGATCACATTGGGTTTGGCG-3′
For *gapdh*
Forward, 5′-TCTTCACCACCATGGAGAAGGCC-3′
Reverse, 5′-GGCAGAAGGGGCGGAGATGA-3′

The copy number of *Arc* ORF was normalized with that of gapdh for each genomic DNA sample.

### Whole-genome sequencing to identify the transgene integration site

A DNA library was constructed from genomic DNA of a homozygous Tg mouse using the TruSeq DNA PCR-Free Library Prep Kit (Illumina) to perform whole-genome sequencing of the sample. The library was sequenced on the HiSeq 2500 sequencer (Illumina), yielding 150-bp paired-end reads. The library adapter sequences at 3′ ends were removed with cutadapt 1.7.1 (Martin, 2011) and low-quality bases (*q*-score < 16) at both ends were then trimmed using a custom script. Pairs containing a read that was shorter than 36 nt were eliminated. As a result, we obtained 124,547,964 read pairs consisting of 36,875,791,529 bases. The 13.5 × FASTQ data were mapped to the mouse reference sequence GRCm38 or mm10 using the MEM algorithm of BWA 0.7.12 (Li and Durbin, 2009). We added the 11,829-bp transgenic sequence (i.e., *Arc* 7k promoter, 5′UTR, *GFP-Arc*, and 3′UTR; Kawashima et al., 2009) to the reference data in advance. If a read was mapped to either the transgene sequence or the *Arc* locus (Chr15:74,669,000–74,682,000), the paired sequences, i.e., along with its counterpart, were extracted to prepare a BAM file. Using SAMtools 1.2 (Li et al., 2009) and Integrative Genomics Viewer 2.3.66 (Robinson et al., 2011), we found many of the counterpart reads were mapped to a region around Chr12:78,138,000. Then, read pairs in which at least one of mates was mapped to an 8-kb region, Chr12:78,134,000–78,142,000, were extracted to prepare another BAM file. Due to apparent lack of mapped reads, a 1.8-kb homozygous deletion was easily detected along with 16 informative read pairs that might span the deletion breakpoints. By aligning these 16 × 2 sequences, two breakpoint sites were determined at single-base resolution. At both sites, sequences of chromosome 12 and the *EGFP-Arc* transgene were connected using 4- and 5-bp shared fragments, respectively. Other integrations were not detected in the whole-genome data. Custom programs, which are available upon request to K.O., were written in Perl and C.

### PCR-based confirmation of the transgene integration site

The transgene integration was detected by PCR using tail DNA with the following specific primers:

Chr12_78M WT primer set 1:
Forward, 5′-AAAGCAATTGTTCACCTGATCTCTGGG-3′
Reverse, 5′-GGTTTCTGTAGGACCTTCACCCACAAG-3′
Chr12_78M WT primer set 2:
Forward, 5′-AGCATCCCTCCAAGGATCATCCCAGTG-3′
Reverse, 5′-TTGGTGGGGGGAGGAAGAGTTCTC-3
Tg junction primer set 1:
Forward, 5′-AAAGCAATTGTTCACCTGATCTCTGGG-3′
Reverse, 5′-CAGGTATCAAGGTGAGTCAGGTCTCCC-3′
Tg junction primer set 2:
Forward, 5′-AGGTTAACCAAGGTCCTACGCCATG-3′
Reverse, 5′-TTGGTGGGGGGAGGAAGAGTTCTC-3

#### Experimental design and statistical analysis

Comparisons between genotypes were done using mice from the breeding colony (mostly male mice with a few females as noted below based on availability at the time of the experiment). Data were analyzed using Prism Version 6.

##### Comparisons of levels of Arc mRNA by genotype (Figure 1L)

Brain sections from WT, hemizygous and homozygous *EGFP-Arc* mice (*n* = 3 males per group) were mounted together on microscope slides and prepared for FISH. Fluorescence intensity was measured over the pyramidal cell layer of CA1. Data were analyzed by one-way ANOVA.

**Figure 1:**
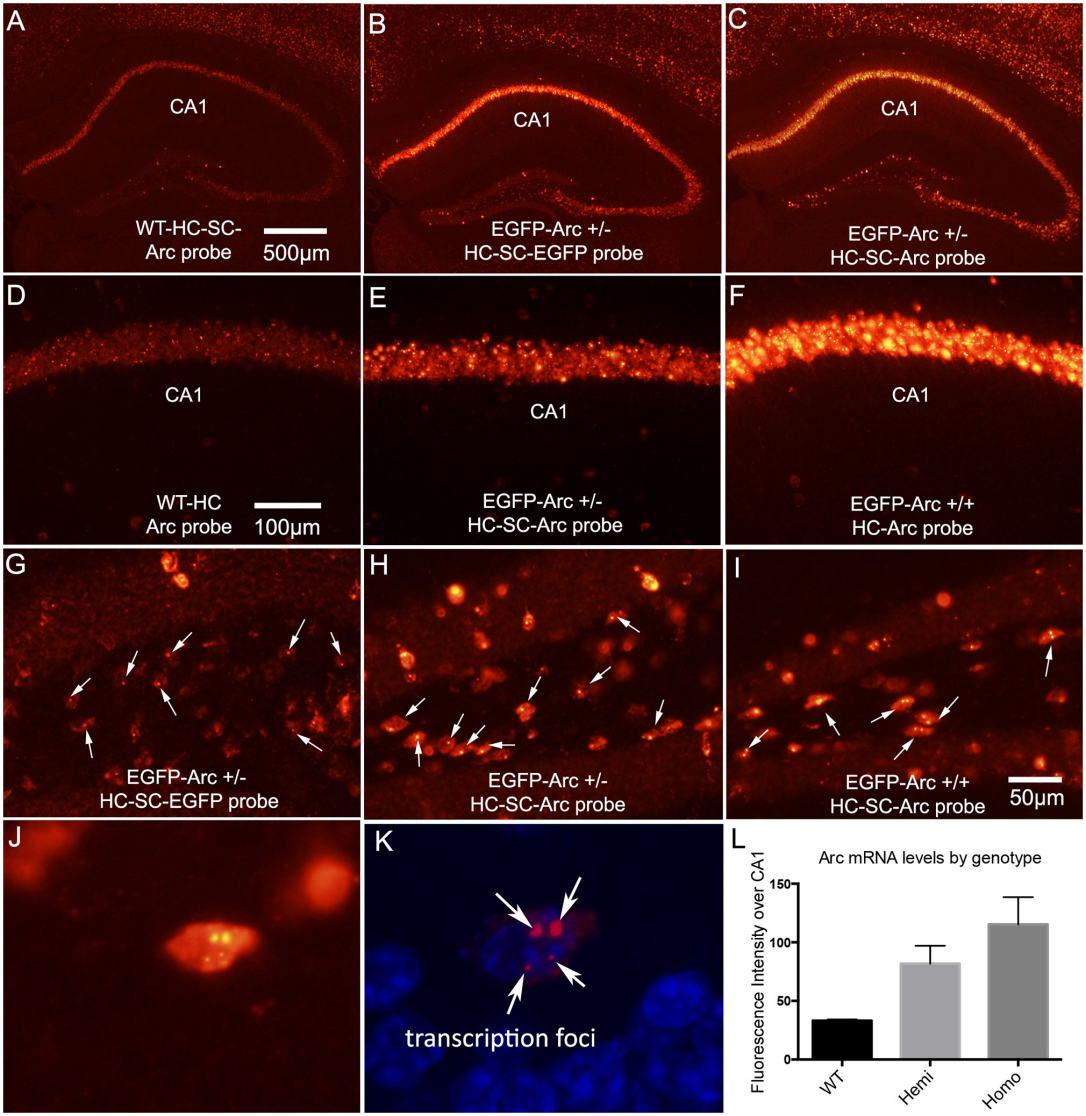
Patterns of expression of endogenous *Arc* vs. *EGFP-Arc* mRNAs. **(A)** Fluorescence *in situ* hybridization (FISH) for *Arc* mRNA in a WT mouse that received I.P. saline and was returned to its home cage for 1 h. HC-SC = home cage, saline control. *Arc* mRNA is detectable in some neurons in the CA1 region of the hippocampus and a few dentate granule cells express *Ar*c at moderate levels. **(B)** Hemizygous *EGFP-Arc* mouse (home cage saline control) hybridized with the *EGFP* probe; **(C)** Hemizygous *EGFP-Arc* mouse (home cage saline control) hybridized with the *Arc* probe; **(D–F)** Higher magnification views of the CA1 region from WT, hemizygous and homozygous transgenic mice. **(G)** Hilar region of the dentate gyrus from hemizygous *EGFP-Arc* mouse (home cage saline control) hybridized with the *EGFP* probe. Arrows indicate fluorescent foci in nuclei; **(H)** Hilar region of the dentate gyrus from hemizygous *EGFP-Arc* mouse (home cage saline control) hybridized with the *Arc* probe; **(I)** Hilar region of the dentate gyrus from homozygous *EGFP-Arc* mouse (home cage saline control) hybridized with the *Arc* probe; **(J)** High magnification view of a neuron in the hilar region from homozygous *EGFP-Arc* mouse (home cage saline control) hybridized with the *Arc* probe. Note two large and two small fluorescent foci in the nucleus. **(K)** Same neuron viewed by confocal microscopy with DAPI shown in blue fluorescence. **(L)** Quantification of fluorescence intensity over CA1 region in mice of different genotypes hybridized with the Arc probe. One-way ANOVA [*F*_(2, 6)_ = 19.83, *p* = 0.0023]; pairwise comparisons by Holm-Sidak multiple comparisons test: WT vs. Hemi, *t* = 3.7, *p* < 0.05; WT vs. Homo, *t* = 6.3, *p* < 0.01; Hemi vs. Homo, *t* = 2.6, *p* < 0.05 Calibration bar in **(A)** = 500 μm and applies to **(A–C)**. Calibration bar in **(D)** = 100 μm and applies to **(D–F)**. Scale bar in **(I)** = 50 μm and applies to **(G–I)**.

##### Quantitative PCR to determine transgene copy number in different genotypes (Figure 2B)

Four WT, 4 hemizygous and 8 homozygous mice were used for PCR. Data were analyzed by one-way ANOVA comparing groups.

**Figure 2:**
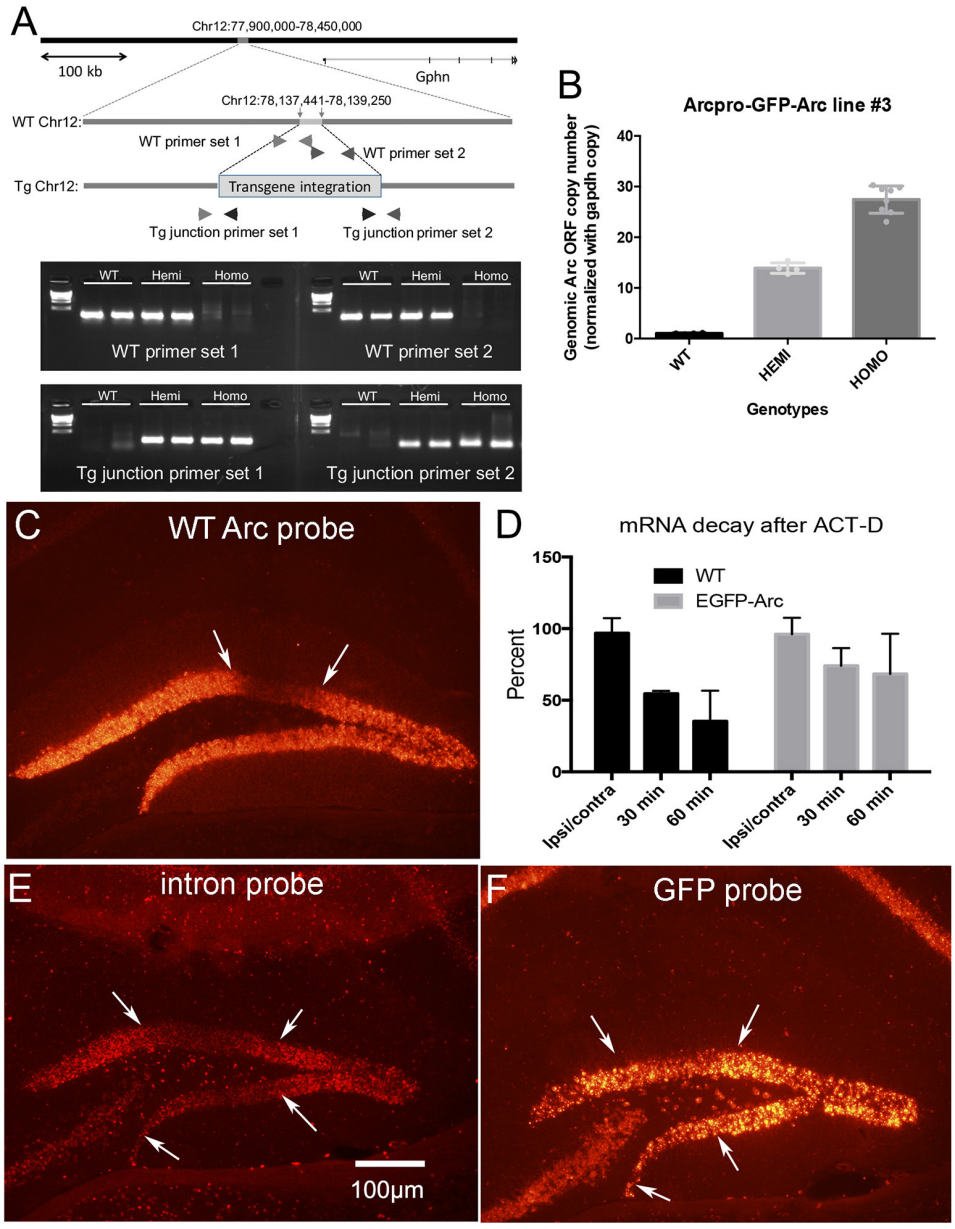
Transgene characterization and assessment of degradation of *Arc* vs. *EGFP-Arc* mRNA following infusion of Act-D into the dentate gyrus. **(A)** Schematic diagram illustrating the genomic integration site of the *EGFP-Arc* transgene at Chromosome 12 of the mouse genome. The most upper panel represents a 550 kb region of mouse Chr12 around the integration site (78,137,4411–78,139,250), showing that the insertion point is 87.4 kb upstream of the nearest gene, Gephyrin (Gphn: 78,226,655–78,684,769). Representative agarose gel patterns at the bottom show PCR-based confirmation of the transgene integration. Locations of the primers are shown in the schematic genomic structures in the middle. **(B)** Quantification of the copy number of the transgene. The copy numbers of *Arc* ORF sequences were estimated in WT, hemizygous, and homozygous mice using qPCR. In **(C–F)** *Arc* transcription was induced by ECS and about 45 min post ECS, Act-D was infused via micropipette positioned in the dentate gyrus. **(C)** *Arc* mRNA levels in wildtype (WT) mice 1 h post-Act-D as revealed by FISH; Act-D injection site is between the white arrows. **(D)** Quantitative assessment of decreases in mRNA levels over time in WT and *EGFP-Arc* transgenic mice. Bars illustrate average fluorescence intensity in the area of the injection as a percent of surrounding areas outside the region of blockade (*n* = 3 mice per time point except for 30 min WT and 60 min *EGFP-Arc* where *n* = 2). **(E)** Pattern of labeling for *Arc* mRNA intron probe in *EGFP-Arc* mouse 1 h. post-Act-D. Note absence of labeling in the area of the Act-D injection site between the white arrows. **(F)** Near-adjacent section from the same mouse shown in **(C)** hybridized with the probe for GFP. Note preservation of labeling in the area between the white arrows indicating abrogation of *EGFP-Arc* mRNA decay. Scale bar = 100 μm.

##### Assessment of mRNA rundown after actinomycin-D infusion in vivo (Figure 2D)

The ratio of fluorescence intensity over the granule cell layer of the dentate gyrus in the area of actinomycin-D infusion vs. the contralateral side of the same section was determined in WT vs. homozygous *EGFP-Arc* mice (*n* = 4 of each genotype without ACT-D infusion, *n* = 2 WT female mice and 4 *EGFP-Arc* male mice at 30 min and *n* = 3 male mice of each genotype at 60 min). Data were analyzed by two-way ANOVA comparing groups vs. time post-ACT-D.

##### Counts of fluorescent puncta in dendritic laminae after ECS (Figure 3E)

Fluorescent puncta were quantified in the molecular layer following ECS in WT vs. *EGFP-Arc* transgenic mice (*n* = 3 mice per group) at 3 locations (outer, middle, and inner molecular layer). Data were analyzed by two-way ANOVA comparing groups and layers.

**Figure 3:**
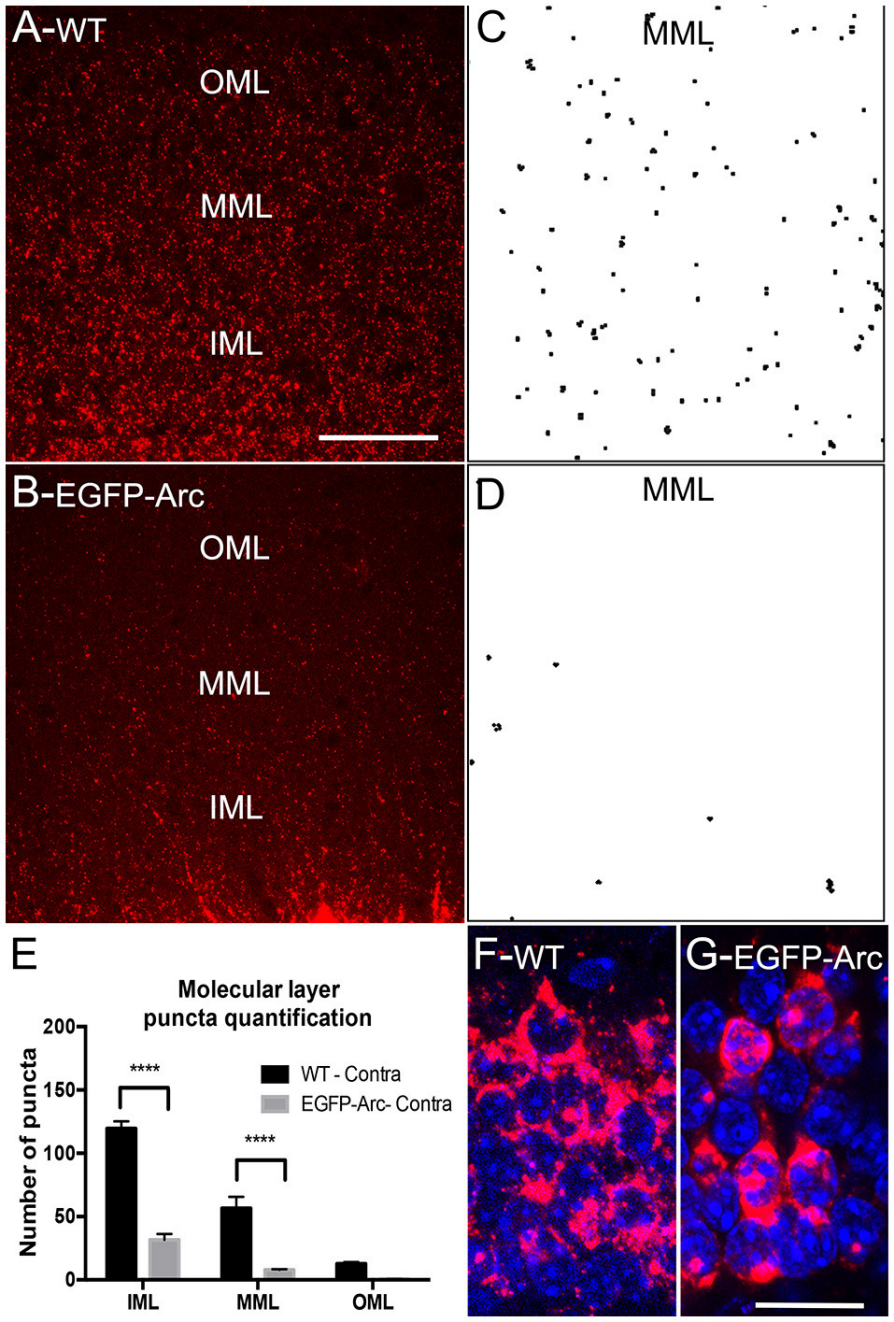
Deficient/delayed dendritic transport of *EGFP-Arc* mRNA after induction by ECS. **(A)** *Arc* mRNA distribution as revealed by FISH following ECS. Note punctate labeling in the dendritic lamina. IML, MML, and OML indicate inner, middle and outer molecular layer, respectively. **(B)** *EGFP-Arc* mRNA distribution in the same case as revealed by FISH for *GFP* mRNA. Note the absence of punctate labeling in the dendritic lamina. **(C)** Collapsed confocal stacks illustrating individual *Arc* mRNA fluorescent puncta from FISH preparations in the middle molecular layer (dendritic laminae of the dentate gyrus). **(D)** Confocal stacks of *EGFP-Arc* mRNA. **(E)** Counts of *Arc* and *EGFP-Arc* mRNA puncta from the inner, middle, and outer molecular layer (IML, MML, and OML, respectively *N* = 3 mice per group at 90–120 min post ECS). Asterisks indicate *p* < 0.0004. **(F)** Confocal stack illustrating *Arc* mRNA fluorescence in granule cell bodies of WT mice; nuclei are stained with DAPI-stained nuclei. **(G)** Confocal stack illustrating *EGFP-Arc* mRNA fluorescence in granule cell bodies of transgenic mice. Scale bar in **(A)** =50 μm and applies to **(A,B)**. Scale bar in **(G)** = 25 μm and applies to **(H,G)**.

##### Quantification of mRNA levels in home cage controls vs. after exploration of a novel environment (Figure 4E)

Groups were: (1) WT-home cage saline, *n* = 2 males; (2) WT-EE saline: *n* = 5 males; (3) *EGFP-Arc*-home cage, *n* = 4 males; (4) *EGFP-Arc*-EE *n* = 5 males. Data on fluorescence intensity over the CA1 pyramidal cell layer were analyzed separately for WT and *EGFP-Arc* by *t*-test.

**Figure 4:**
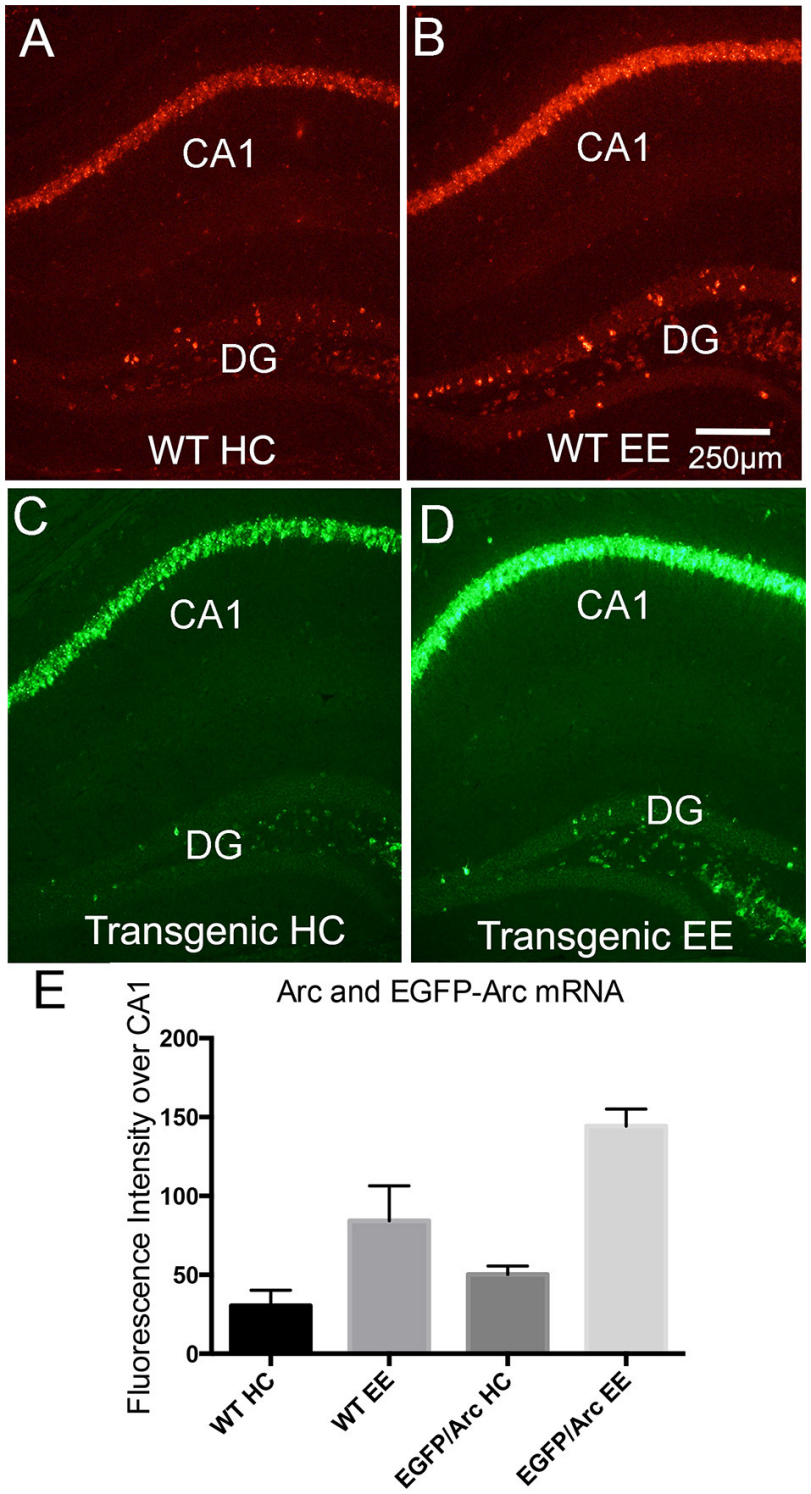
Induction of *Arc* and *EGFP-Arc* mRNAs by exploration of a novel environment. **(A)** Fluorescence *in situ* hybridization (FISH) for *Arc* mRNA in a WT mouse that was anesthetized immediately after being removed from its home cage (WT HC). **(B)** FISH labeling for *Arc* mRNA in a WT mouse that was allowed to explore a novel enriched environment for 1 h (WT EE). **(C)** FISH *for EGFP-Arc* mRNA (FITC detection) in an *EGFP-Arc* hemizygous mouse that was anesthetized immediately after being removed from its home cage (Transgenic HC). **(D)** FISH for *EGFP-Arc* mRNA (FITC detection) in an *EGFP-Arc* hemizygous mouse that was allowed to explore a novel enriched environment for 1 h (Transgenic EE). **(E)** Quantification of *Arc* and *EGFP-Arc* mRNAs as a result of experience. Sections from all mice were hybridized at the same time for either *Arc* or *GFP mRNA*, and levels of fluorescence over the pyramidal cell layer of CA1 were quantified (as in Figure 1L). Scale bar in **(B)** = 250 μm.

##### Quantification of Western blot (Figure 5H)

Samples from *EGFP-Arc* tg/tg (*n* = 4 females and 1 male) and WT controls (*n* = 3 females and 1 male) were run on the same Western blot and fluorescence signal intensity in Arc and Arc-GFP bands was normalized to the beta actin loading control. Data were analyzed by one-way ANOVA.

**Figure 5:**
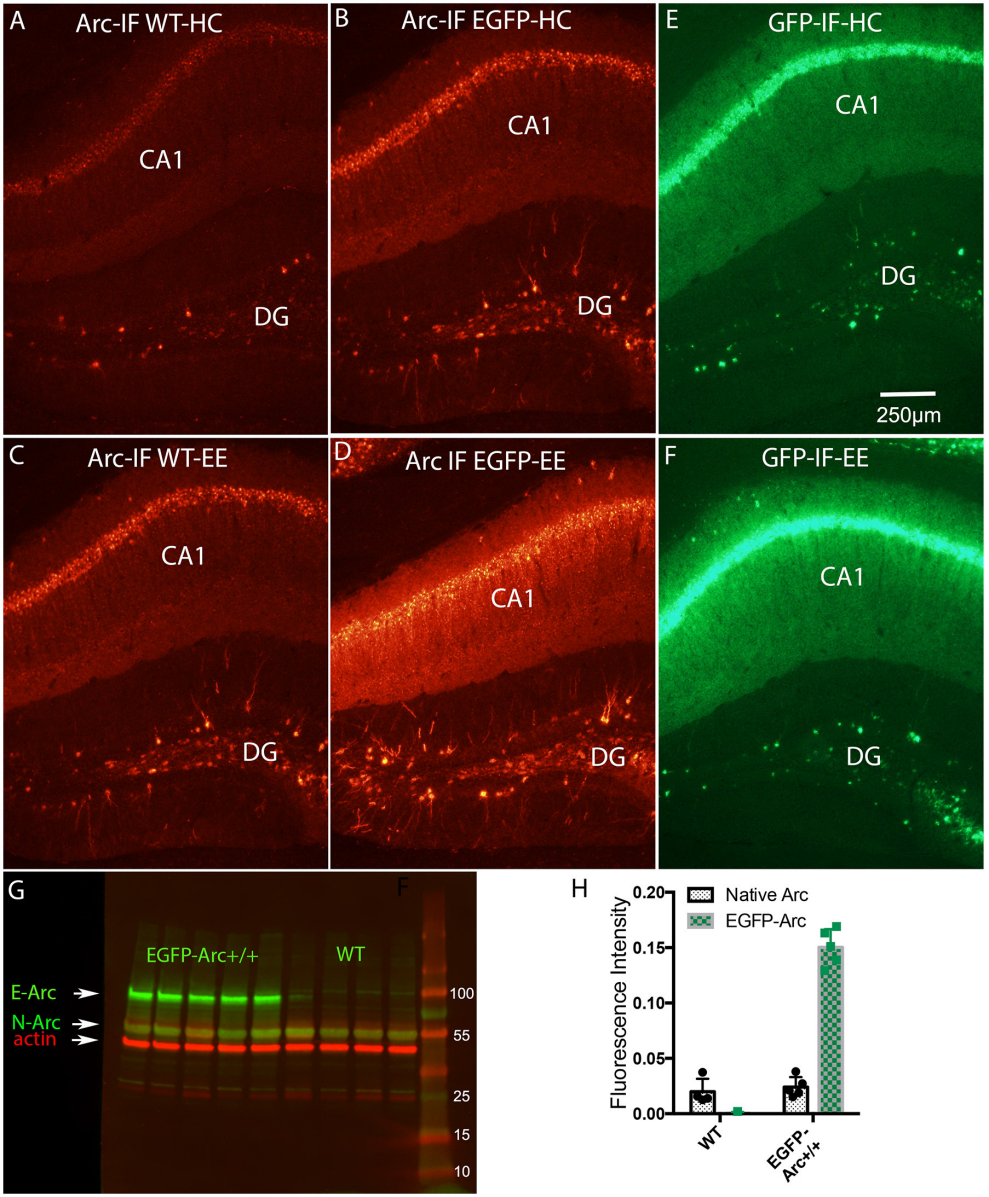
Induction of expression of *Arc* and *EGFP-Arc* mRNAs by exploration of a novel environment is accompanied by increased expression of Arc and EGFP-Arc proteins. **(A)** Immunofluorescence for Arc protein in a WT mouse that was anesthetized immediately after being removed from its home cage (Arc-IF WT-HC). **(B)** Immunofluorescence for Arc protein in an EGFP-Arc transgenic mouse that was that was anesthetized immediately after being removed from its home cage (Arc-IF EGFP-HC). Note higher levels of labeling for Arc protein in the *EGFP-Arc* transgenic mouse (Arc-IF EGFP-HC). **(C)** Immunofluorescence for Arc protein in a WT mouse that was allowed to explore a novel enriched environment for 1 h (Arc-IF WT-EE). Note substantial induction Arc protein expression. **(D)** Immunofluorescence for Arc protein in an *EGFP-Arc* transgenic mouse that was allowed to explore a novel enriched environment for 1 h (Arc-IF EGFP-EE). **(E)** Immunofluorescence for GFP in an EGFP transgenic mouse that was that was anesthetized immediately after being removed from its home cage (GFP-IF-HC). **(F)** Immunofluorescence for GFP in an *EGFP-Arc* transgenic mouse that was allowed to explore a novel enriched environment for 1 h (GFP-IF-EE). **(G)** Western Blot analysis of Arc protein in WT (*n* = 4 mice) and *EGFP-Arc* transgenic mice (*n* = 5). Green fluorescence: Arc protein ß-actin: red fluorescence. E-ARC: band of fluorescence for EGFP-Arc in the transgenic. N-Arc: band of fluorescence for endogenous Arc protein. **(H)** Quantification of the levels of fluorescence in the N-Arc and E-Arc bands (two-way ANOVA, *p* < 0.0001). Scale bar in **(E)** = 250 μm.

##### Neurophysiology (Figures 6, 7)

As noted in the Results, localization of Arc at activated synapses in the ECS-ECStim paradigm, which is highly reliable in rats, occurs less reliably in mice. Accordingly, data on *Arc* mRNA distribution across the molecular layer are quantified for individual mice, but are not analyzed statistically.

**Figure 6:**
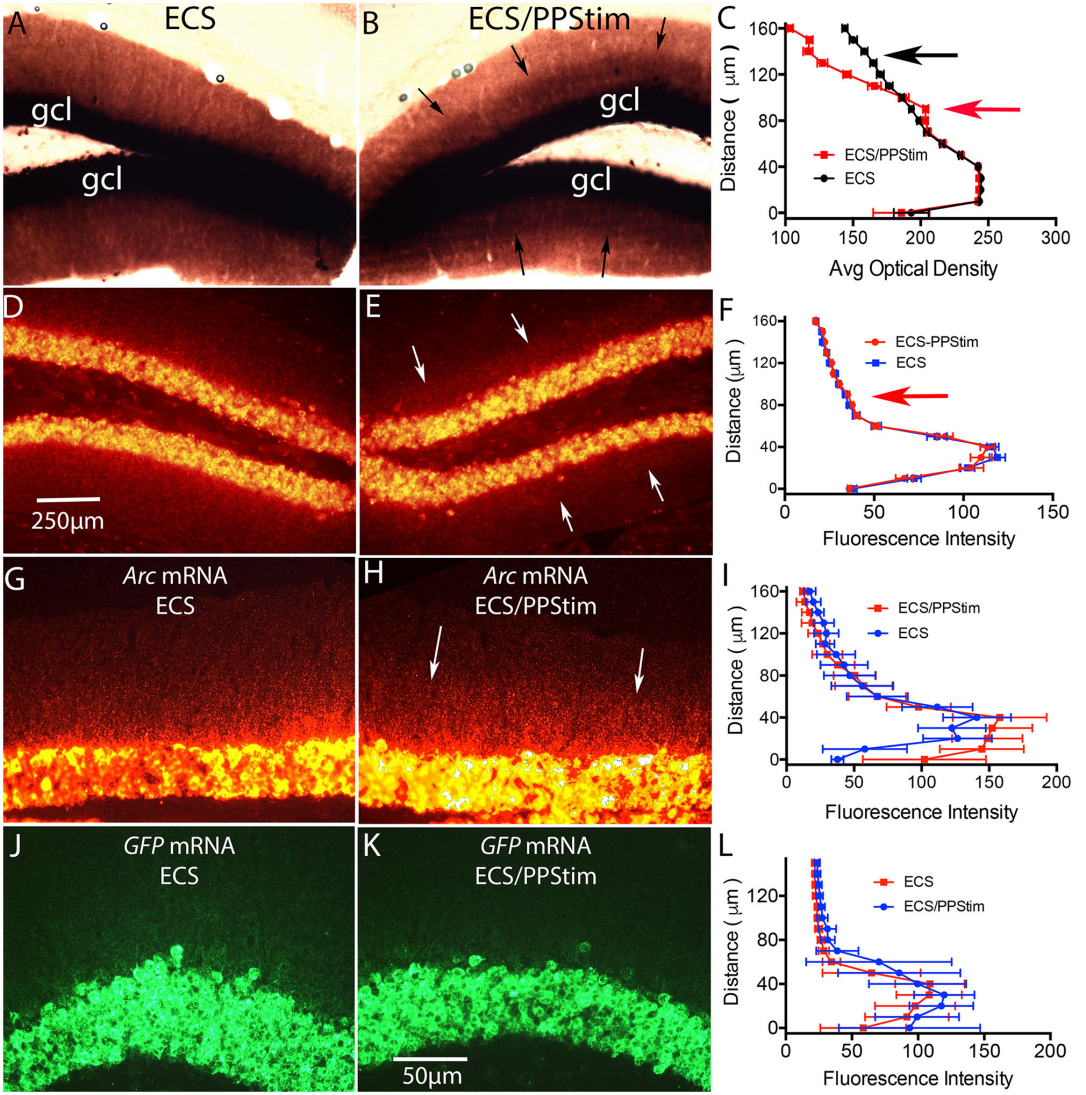
Assessment of localization of *Arc* and *EGFP-Arc* mRNA at active synapses: *ECS-Perforant Path stimulation* paradigm (termed ECS/PPStim). Mice received a single ECS to induce *Arc* time is allowed for the mRNA to move throughout dendrites (1 h), then high frequency stimulation (HFS) is delivered to the perforant path, which causes newly-synthesized *Arc* mRNA to localize selectively in the activated dendritic lamina (Steward and Worley, 2001). Accumulation of *Arc* mRNA in the activated dendritic lamina is accompanied by disappearance from non-activated portions of the dendrite leading to a selective band of labeling in the activated lamina. **(A)** Chromogen-based ISH for *Arc* mRNA after ECS; WT C57Bl/6 mouse. **(B)** Distinctive laminar pattern of labeling on the side that received perforant path stimulation. Arrows indicate the distinctive boundary between the activated lamina and the outer molecular layer. **(C)** Quantification of optical density (OD) across the granule cell layer (GCL) and dendritic laminae. The red arrow indicates the higher level of labeling in the activated zone; the black arrow indicates the outer molecular layer where levels of labeling are decreased on the stimulated side. **(D)** FISH for *Arc* mRNA after ECS in WT mouse. **(E)** Distinctive laminar pattern of labeling on the side that received perforant path stimulation. Arrows indicate the boundary between the activated lamina and the outer molecular layer. **(F)** Quantification of fluorescence intensity across the granule cell layer (GCL) and dendritic laminae. **(G)** *Arc* mRNA distribution in a tg/tg transgenic mouse after ECS as revealed by FISH for *Arc* mRNA. **(H)** *Arc* mRNA distribution on the side that received perforant path stimulation (PPStim). **(I)** Quantification of fluorescence intensity across the granule cell layer and dendritic laminae from panels **(G,H)**. **(J,K)** *EGFP-Arc* mRNA distribution in the same *EGFP-Arc* transgenic mouse after ECS as revealed by FISH for *GFP* mRNA. **(L)** Quantification of fluorescence intensity across the granule cell layer and dendritic laminae from panels **(J,K)**. Scale bar in **(D)** = 250 μm and applies to **(A,B,D,E)**. Scale bar in **(K)** = 50 μm and applies to **(G,H,J,K)**.

**Figure 7:**
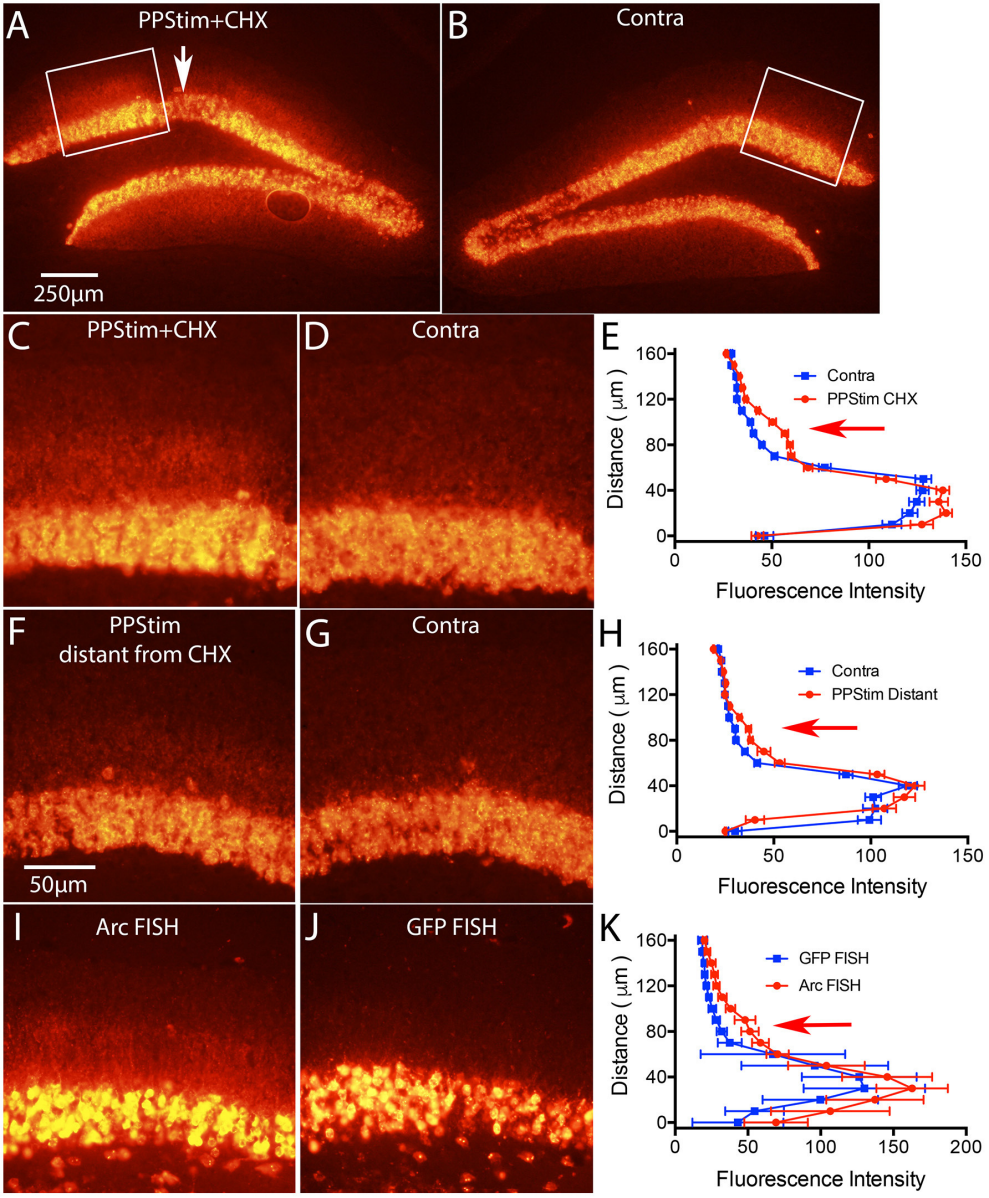
Local infusion of CHX leads to increases in *Arc* mRNA levels in the area of protein synthesis blockade, consistent with translation-dependent mRNA decay. Mice received a single ECS to induce *Arc* and 1 h later were prepared for neurophysiology with a recording micropipette filled with CHX. Then high frequency stimulation (HFS) was delivered to the perforant path. **(A)** *Arc* mRNA distribution as revealed by FISH in the area of CHX infusion with perforant path stimulation (PPStim). Arrow indicates path of CHX-containing micropipette. **(B)** *Arc* mRNA distribution on the contralateral side (ECS only). White boxes indicate the areas illustrated in **(C,D)**. **(C,D)** Higher magnification views of *Arc* mRNA distribution across the cell layer and dendritic layers in the area of CHX infusion and on the contralateral side of the same section. **(E)** Graph of fluorescence intensity across the cell layers and dendritic layers from images in **(C,D)**. Red arrow indicates the band of *Arc* mRNA in the activated dendritic lamina. **(F,G)** *Arc* mRNA distribution across the cell layer and dendritic layers in a section distant from area of CHX infusion and on the contralateral side of the same section. **(H)** Graph of fluorescence intensity across the cell layers and dendritic layers from images in **(F,G)**. **(I,J)** illustrate the distribution of *Arc* mRNA and *GFP* mRNA, respectively from an experiment in which mice received a single ECS to induce *Arc* and were prepared for neurophysiology immediately with a recording micropipette filled with CHX. Then high frequency stimulation (HFS) was delivered to the perforant path. Note accumulation of *Arc* mRNA in the activated lamina in **(I)** and the absence of *GFP* mRNA in dendrites in **(J)**. **(K)** Graph of fluorescence intensity across the cell layers and dendritic layers from images in **(I,J)**. Scale bar in **(A)** = 250 μm and applies to **(A,B)**. Scale bar in **(F)** = 50 μm and applies to **(C,D)**, **(F,G)**, and **(I,J)**.

##### Quantification of mRNA levels in home cage controls vs. after 1 h exploration of a novel environment with and without CHX treatment (Figures 8M,N)

Groups were: (1) WT-home cage saline, *n* = 2 males; (2) WT-home cage CHX, *n* = 4 males, (3) WT-EE saline: *n* = 5 males; (4) WT-EE CHX: *n* = 3 males, 2 females; (5) *EGFP-Arc*-home cage saline, *n* = 4 males; (6) *EGFP-Arc*-home cage CHX, *n* = 4 males; (7) *EGFP-Arc*-EE saline *n* = 5 males; (8) *EGFP-Arc*-EE CHX *n* = 5 males. Data on fluorescence intensity over the CA1 pyramidal cell layer were analyzed by two-way ANOVA comparing treatment (saline vs. CHX) and experience (home cage vs. EE). Data from saline-treated control mice are from Figure 4.

**Figure 8:**
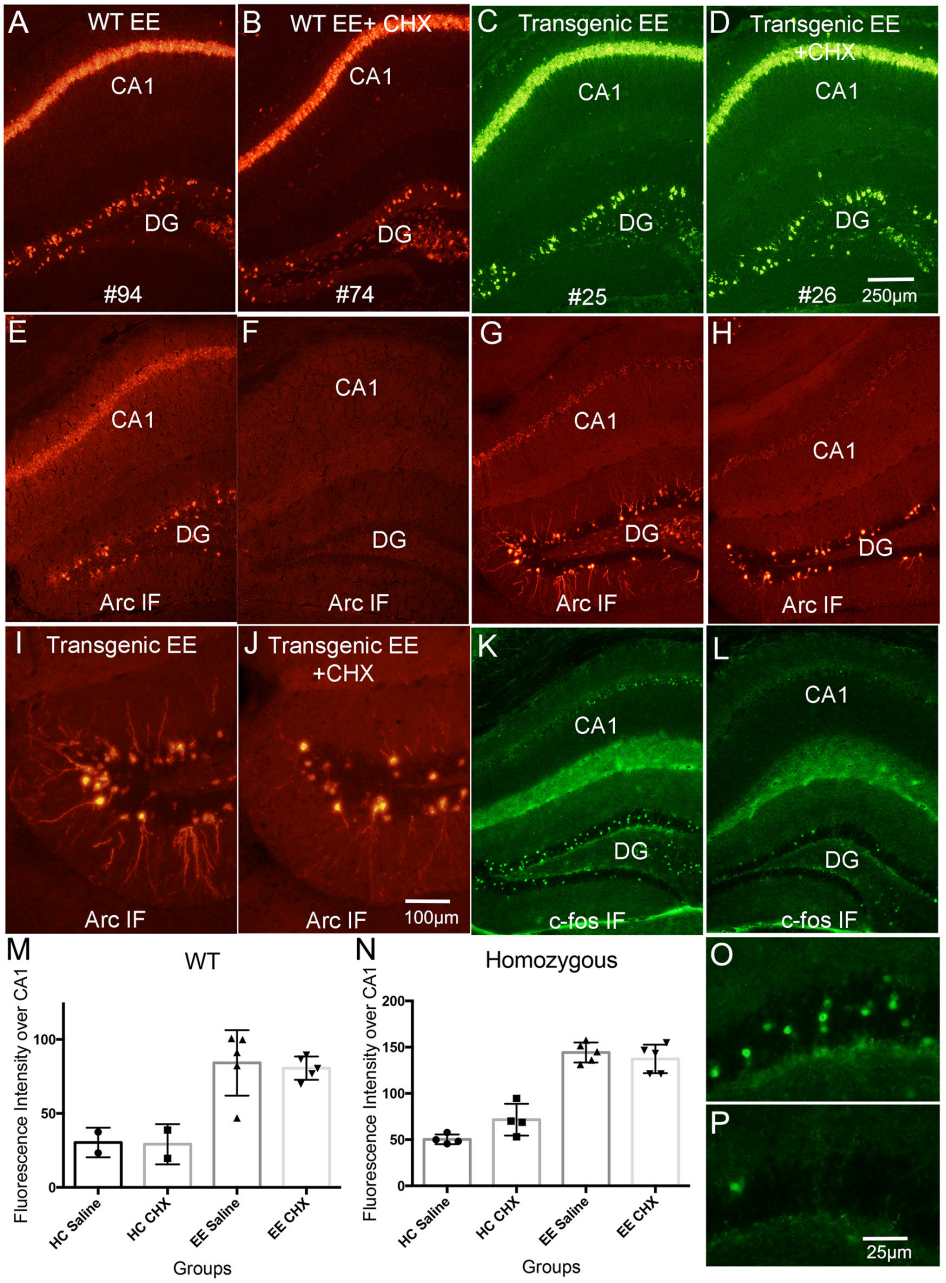
Systemic delivery of CHX does not lead to increases in *Arc* mRNA in awake behaving mice. WT and Transgenic mice were allowed to explore a novel enriched environment with and without CHX treatment to inhibit protein synthesis. **(A)** Fluorescence *in situ* hybridization (FISH) for *Arc* mRNA in a WT mouse that received saline (WT EE). **(B)** Fluorescence *in situ* hybridization (FISH) for *Arc* mRNA in a WT mouse that received CHX (WT EE+CHX). **(C)** Fluorescence *in situ* hybridization (FISH) for *Arc* mRNA for *EGFP-Arc* mRNA (FITC detection) in a tg/tg transgenic mouse that received saline (Transgenic EE). **(D)** Fluorescence *in situ* hybridization (FISH) for *Arc* mRNA (FITC detection) in a tg/tg transgenic mouse that received CHX (Transgenic EE+CHX). **(E,F)** Immunofluorescence for Arc protein in the WT mice illustrated in **(A,B)**. Note absence of Arc IF in the WT mouse treated with CHX **(F)**. **(G,H)** Immunofluorescence for Arc protein in the tg/tg transgenic mice illustrated in **(C,D)**. Note persistence of Arc IF in the *EGFP-Arc* transgenic mouse treated with CHX **(H)**. **(I,J)** Higher magnification views of Arc positive granule cells in the dentate gyrus from the sections shown in **(G,H)**. Note some decrease in immunofluorescence of dendrites in **(J)**. **(K,L)** Immunofluorescence for c-Fos protein in the transgenic mice illustrated in **(G,H)** to confirm rundown of c-Fos protein in transgenic mice after CHX treatment. **(M)** Quantification of fluorescence intensity for *Arc* mRNA over CA1 for the different groups of WT mice. **(N)** Quantification of fluorescence intensity for *Arc* mRNA over CA1 for the different groups of tg/tg transgenic mice. *EGFP-Arc* mRNA levels were somewhat elevated in transgenic mice that received CHX vs. saline and were returned to their home cage, but differences were not statistically significant (*t* = 2.71, *df* = 6, *p* = 0.412). **(O,P)** Higher magnification views of c-Fos positive granule cells in the dentate gyrus from the sections shown in **(G,H)**. Scale bar in **(D)** = 250 μm and applies to **(A–L)**. Scale bar in **(J)** = 100 μm and applies to **(I,J)**. Scale bar in *P* = 25 μm and applies to **(O,P)**.

## Results

### Total *Arc* mRNA (*EGFP-Arc* + endogenous *Arc* mRNA) is overexpressed in *EGFP-Arc* transgenic mice

*Arc* is expressed as an immediate early gene (IEG); under resting conditions, mRNA levels are low or non-detectable in most neurons in the cortex and hippocampus, whereas transcription is strongly induced by learning experiences or other events that cause strong synaptic activation. Although *Arc* is expressed in neurons throughout the forebrain, we focus here on patterns of *Arc* expression in the hippocampus and dentate gyrus because these illustrate general rules regarding regulation of *Arc* expression. Figure 1A illustrates fluorescence *in situ* hybridization (FISH) for *Arc* mRNA in a WT mouse that was one of the “saline controls” for the study described below involving injections of cycloheximide (CHX); as such, this mouse received an intra-peritoneal (IP) injection of saline and was returned to its home cage for 1 h before being anesthetized. *Arc* mRNA is detectable in some neurons in the CA1 region of the hippocampus and a few dentate granule cells express *Ar*c at moderate levels. As will be seen below, this extent of expression is somewhat higher than seen in mice that were anesthetized immediately upon removal from their home cage without prior saline injections, reflecting some activation as a result of the handling and IP injection 1 h prior to euthanasia.

To assess whether expression of *EGFP-Arc* is expressed in a similar way as endogenous *Arc*, we used hemizygous mice with one allele of *EGFP-Arc* transgene, and assessed transgene expression using cRNA probes specific for *EGFP-Arc* mRNA. As illustrated in Figure 1B, the overall pattern of expression in the hippocampus was comparable to endogenous *Arc* mRNA. If *EGFP-Arc* mRNA is not subject to the same decay mechanisms as endogenous *Arc* mRNA, then overall levels of labeling for *EGFP-Arc* should be higher than for endogenous *Arc* in WT mice. Consistent with this prediction, when sections from hemizygous mice were also hybridized using the probe for endogenous *Arc*, which recognizes both *Arc* and *EGFP-Arc* mRNAs because the full-length coding sequence of *Arc* is present in the transgene, levels of labeling were much higher than for endogenous *Arc* in WT mice (Figure 1C). It is important to note that the images in Figures 1A,C are from sections from WT and hemizygous mice that were processed together in the same *in situ* hybridization run using the same cRNA probe.

Comparisons of levels of *Arc* mRNA in WT, hemizygous and homozygous *EGFP-Arc* mice revealed that levels of labeling for *Arc* mRNA were higher in homozygous *EGFP-Arc* than in hemizygous, and both were higher than WT. This can be appreciated by comparing levels of labeling in the CA1 region in WT (Figure 1D), hemizygous (Figure 1E), and homozygous *EGFP-Arc* mice (Figure 1F). Differences in labeling by genotype were confirmed by measuring fluorescence intensity over the CA1 region in 3 mice per genotype (Figure 1L). One-way ANOVA, revealed overall significance [*F*_(2, 6)_ = 19.83, *p* = 0.0023] and pairwise *post-hoc* comparisons by the Holm-Sidak multiple comparisons test indicated significant differences between all 3 genotypes (for details on statistics, see figure legend). Although elevated levels of *EGFP/Arc* mRNA are consistent with impaired mRNA decay, it is important to note that overall *Arc* transcription is also elevated due to the presence of both native and *EGFP-Arc* and the fact that there are multiple copies of the transgene (see below).

We note here that there is little detectable labeling for *EGFP-Arc* mRNA in dendritic lamina in CA1 despite high levels of labeling over pyramidal cell bodies. We initially suspected that this might be due to the fact that dendritic labeling is most prominent after *Arc* is induced by some stimulus. As documented below, however, there is little detectable dendritic labeling for *EGFP-Arc* mRNA when *Arc* is massively induced in dentate granule cells by ECS.

### Visualization of *EGFP-Arc* mRNA transcription foci

With fluorescence *in situ* hybridization, mRNAs appear as discrete fluorescent puncta, and active transcription foci appear as distinct spots in neuronal nuclei. For example, transcription foci are evident in neurons in the CA1 region in the image from WT mice in Figure 1D. We noticed that when using a probe specific for *EGFP-Arc* in hemizygous *EGFP-Arc* mice, there were single distinct fluorescent foci in the nuclei of many neurons (Figure 1G). This is a higher magnification view of the hilar region of the dentate gyrus in the section shown in Figure 1B. We interpret these foci as transcription sites for *EGFP-Arc*, and simple face validation of this interpretation is that two foci are evident in nuclei of neurons in sections from tg/tg mice that were hybridized with the probe for *EGFP-Arc* (not shown, but see next). Interestingly, when sections from hemizygous *EGFP-Arc* mice were hybridized with the probe for endogenous Arc, which recognizes both *EGFP-Arc* and endogenous *Arc*, single fluorescent foci were evident in the nucleus of many neurons (Figure 1H; each arrow points to a neuron with a single focus). In contrast, 2 foci were evident in the nuclei of homozygous *EGFP-Arc* mice hybridized with the probe for endogenous *Arc* (Figure 1I; each arrow points to a neuron with two foci).

The fact that there is one large labeled focus in nuclei per allele of the transgene suggests higher levels of labeling of the *EGFP-Arc* transcription site vs. the two transcription sites for WT *Arc*. Support for this interpretation comes from the fact that in homozygous transgenic mice, neurons could be seen with two large and two small fluorescent nuclear foci. This can be seen with regular fluorescence microscopy (Figure 1J), but is even more evident by confocal microscopy (Figure 1K). Because the number of large foci varies by genotype, it is highly likely that the two large foci are transcription foci for *EGFP-Arc* mRNA and the two small foci are transcription foci for WT *Arc mRNA*.

Four spatially-separate loci are consistent with the fact that whole-genome sequencing in the transgenic line used in this study revealed that the endogenous *Arc* gene and *EGFP*-*Arc* transgene are on different chromosomes; the endogenous *Arc* gene is present on Chr 15 while the *EGFP-Arc* transgene is integrated at Chr 12. The exact integration site of the transgene was identified at Chr12:78,137,441 and 78,139,250, resulting in a deletion of 1,808 bp. Analyses with UCSC-Genome Browser and PCR revealed that there are no disrupted genes by the integration of transgene at this site (Figure 2A).

One possible explanation for the higher levels of labeling at *EGFP-Arc* transcription sites is that multiple copies of the transgene integrate in tandem into the locus. To assess this, we used quantitative PCR to estimate copy number (see section Methods), which revealed that that there are approximately 15 copies of the transgene per locus [Figure 2B, One-way ANOVA *F*_(2, 13)_ = 233.7, *p* < 0.0001]. Thus, the higher levels of labeling at the *EGFP-Arc* transcription foci is most likely due to integration of multiple copies of the transgene. Other contributions cannot be excluded, including: (1) *EGFP-Arc* may be transcribed at a higher level than endogenous *Arc* in the same individual nucleus driven by its own 7 kb Arc promoter. (2) *EGFP-Arc* pre-mRNA does not contain the introns present in endogenous *Arc* pre-mRNA, and thus may be retained at transcription foci for longer than endogenous *Arc* that undergoes splicing. (3) The presence of the coding sequence of *EGFP* in tandem with the coding sequence of *Arc* may interfere with intra-nuclear processing, causing the *EGFP-Arc* transcript to be retained near the transcription site. Whatever the explanation, the large foci provide a convenient and reliable means to genotype mice by *in situ* hybridization.

### *EGFP-Arc* mRNA is degraded more slowly than endogenous *Arc* mRNA

To determine the time course of degradation of *EGFP-Arc* mRNA vs. endogenous *Arc* mRNA, we assessed rundown of mRNA levels after transcriptional block with Act-D. *Arc* transcription was induced in homozygous transgenic and WT mice by delivering an electroconvulsive seizure (ECS), mice were prepared for *in vivo* neurophysiology and a recording micropipette containing Act-D was stereotaxically positioned in the dentate gyrus at approximately 1 h post-ECS. After 30 and 60 min, brains were harvested for FISH.

After a single ECS, *Arc* transcription is strongly induced in dentate granule cells and transcriptional activation persists for hours. This is evidenced by continued high levels of labeling for *Arc* mRNA in dentate granule cells. In WT mice, local infusion of Act-D led to progressive decreases in *Arc* mRNA levels in an area about 300–500 μm in diameter surrounding the micropipette (Figure 2C; area of blockade is between the arrows). To quantify the time course of mRNA decay, we analyzed *Arc* mRNA fluorescence intensity in the region of Act-D infusion and in the surrounding regions of the dentate gyrus and expressed this as a ratio (2 mice at 30 min and 3 mice at 60 min). At each time point, *Arc* mRNA levels decreased to about half of the level 30 min prior (Figure 2D). This is consistent with previous determinations of the time course of *Arc* mRNA decay after Act-D treatment of neurons in culture, which indicate a half life for *Arc* mRNA of about 45 min (Rao et al., 2006).

When the same experiment was done with homozygous *EGFP-Arc* mice and sections were hybridized for *EGFP* mRNA, it was difficult to locate an area of diminished labeling due to Act-D. To define the area of transcriptional blockade, sections from *EGFP-Arc* mice were hybridized with a probe specific for the intron in endogenous *Arc* mRNA. As expected, the area of transcriptional blockade was revealed by absence of labeling for the intron probe in an area surrounding the Act-D filled micropipette (Figure 2E). Based on this identification of the region of transcriptional blockade, we hybridized nearby sections (usually adjacent sections) for *EGFP* mRNA (Figure 2F), and quantified levels of labeling in the area of transcriptional blockade vs. nearby regions (4 mice at 30 min; 3 mice at 60 min). This analysis confirmed delayed rundown of *EGFP-Arc* mRNA levels with Act-D (Figure 2D, two-way ANOVA Group difference [*F*_(1, 14)_ = 5.289, *p* = 0.0374], effect of time [*F*_(1, 14)_ = 14.12, *p* = 0.0004]; by 60 min, levels of labeling for *EGFP-Arc* mRNA remained about 75% of the levels at sites outside the area of blockade.

### Impaired dendritic delivery of *EGFP-Arc* mRNA after induction by seizures

In our assessment of *Arc vs. EGFP-Arc* mRNA levels after ECS, it was again evident that levels of labeling in dendrites appeared lower for *EGFP-Arc* mRNA than for endogenous *Arc* mRNA (compare Figure 3A: *Arc* WT with Figure 3B: *EGFP-Arc*). To assess this, sections were imaged by confocal microscopy in order to resolve individual fluorescent puncta in the dendritic laminae of the dentate gyrus (Figures 3C,D). We then counted the number of puncta in the inner, middle, and outer molecular layer (IML, MML, and OML, respectively *n* = 3 mice per group at 90–120 min post ECS). Quantitative analyses confirmed that despite strong induction of transcription, there were far fewer dendritic *EGFP-Arc* mRNA puncta than *Arc* mRNA puncta in WT mice [Figure 3E, 2-way ANOVA, difference between groups: *F*_(1, 4)_ = 119.9, *p* = 0.0004, difference between layers: *F*_(2, 8)_ = 130.7, *p* < 0.0001]. These results indicate impaired dendritic delivery of *EGFP-Arc* mRNA to distal dendritic regions, even when *Arc* mRNA transcription is strongly induced.

One possible explanation for impaired dendritic delivery is impaired nucleocytoplasmic transport of newly-synthesized *EGFP-Arc* mRNA. To explore this possibility, we used confocal microscopy to image fluorescence from FISH and nuclei of dentate granule cells stained with DAPI to assess whether newly-synthesized *EGFP-Arc* mRNA entered the cytoplasm. Confocal images in in WT mice revealed high levels of cytoplasmic labeling for *Arc* mRNA consistent with efficient nucleocytoplasmic transport (Figure 3F). In transgenic mice, *EGFP-Arc* mRNA was also clearly present in cytoplasm (Figure 3G). It is noteworthy that *Ar*c mRNA in WT mice is present in discrete granules whereas *EGFP-Arc* mRNA appears in globs, inviting the speculation that there may be a deficiency in packaging *EGFP-Arc* mRNA into granules or in granule trafficking.

### *EGFP-Arc* transcription is regulated by experience in a manner similar to endogenous *Arc*

*Arc* mRNA transcription is induced by learning experiences, and a simple paradigm to demonstrate this is to allow animals to explore a novel toy-filled enriched environment (EE), which in WT mice is accompanied by robust induction of *Arc* transcription (Farris et al., 2014). Figure 4A illustrates fluorescence *in situ* hybridization for *Arc* mRNA in a WT mouse that was anesthetized immediately after being removed from its home cage and Figure 4B illustrates the labeling for *Arc* in a WT mouse that was allowed to explore a novel environment for 1 h. As previously described in rats (Farris et al., 2014), this learning experience led to increases in *Arc* mRNA levels in neurons in the CA1 region of the hippocampus, and increases in the number of *Arc*-positive dentate granule cells. For quantitative analyses, fluorescence levels were quantified over the pyramidal cell layer of CA1 as in Figure 1L (Figure 4E). Endogenous *Arc* mRNA levels were about 3-fold higher after 1 h of exploration (*n* = 2HC, 5EE, *t* = 3.169, *p* = 0.0248). *EGFP-Arc* mRNA was expressed with similar dynamics; Figure 4C illustrates the pattern of expression of *EGFP-Arc* mRNA in a home cage control hemizygous mouse and Figure 4D illustrates striking increases in *EGFP-Arc* mRNA levels in a hemizygous mouse that was allowed to explore a novel environment for 1 h. In Figures 4C,D, *EGFP-Arc* mRNA was detected using FITC, which provides a convenient color code for illustrating *EGFP-Arc* vs. *Arc* mRNAs. Levels of fluorescence for *EGFP-Arc* mRNA as assessed by hybridization using the probe for GFP was elevated to about the same extent as endogenous *Arc* mRNA (*n* = 4HC, 5EE, *t* = 15.78, *p* < 0.0001).

It should be noted that the “home cage controls” illustrated in Figure 4A were transported in their home cage from the vivarium to the testing room along with the mice that were exposed to the novel environment, which included transport in an elevator, and then the mice were euthanized. Probably because of this experience, levels of labeling are higher over the pyramidal cell layers and there are more *Arc*-positive granule cells in the mouse illustrated in Figure 4A than in the mouse illustrated in Figure 1A, which was euthanized immediately after removal from its home cage in the vivarium.

### Total Arc protein levels (endogenous Arc + EGFP-Arc) are elevated in transgenic mice and increase further as a result of a learning experience

To assess whether differences in levels of expression of *Arc* and *EGFP-Arc* mRNAs were paralleled by differences in the levels of the respective proteins, sections were immunostained using antibodies against Arc. In WT mice, immunofluorescence for Arc protein in home cage controls revealed patterns of expression that were similar to what has been described in previous studies (Figure 5A). Levels of labeling were low over the cell body laminae of the hippocampus. Most dentate granule cells were unstained, but there were a few labeled granule cells scattered through the granule cell layer, especially in the dorsal blade. As noted above, EGFP-Arc protein contains the full Arc sequence, so antibodies against Arc also recognize EGFP-Arc protein, so immunofluorescence indicates expression of total Arc protein (endogenous Arc + EGFP-Arc). As with the mRNAs, levels of expression of Arc and EGFP-Arc proteins were much higher under basal (home cage) conditions in EGFP-Arc transgenic mice (Figure 5B). Especially noteworthy was the high level of immunofluorescence throughout the dendritic laminae of CA1 and prominent staining of dendrites of dentate granule cells.

In WT mice, 1 h of exploration in an enriched environment led to robust increases in Arc immunofluorescence in hippocampal subfields, especially in CA1, and increases in the number of Arc-positive granule cells (Compare Figure 5C with Figure 5A). There was also strong induction of expression of total Arc protein (endogenous Arc + EGFP-Arc) in transgenic mice (Compare Figure 5D with Figure 5B). It was noteworthy that by the end of the 1 h in the enriched environment, total Arc protein levels increased in both cell bodies and throughout dendrites. This was especially evident in CA1, where increases in immunofluorescence were seen even in the zone containing the most distal dendrites of CA1 pyramidal cells (*stratum oriens* and *stratum lacunosum-moleculare)*.

Sections from the same transgenic mice were also immunostained using the antibody for GFP so as to detect the level and distribution of EGFP-Arc protein only. Overall, patterns of labeling for EGFP mirrored the patterns for total Arc protein; especially noteworthy was that levels of immunofluorescence for GFP were high over the dendritic laminae of the hippocampus under resting conditions (Figure 5E) and increased further after 1 h in the enriched environment (Figure 5F).

In order to estimate the extent to which total Arc protein was elevated in EGFP-Arc transgenic mice, cortical tissue from WT (*n* = 4) and homozygous transgenic (*n* = 5) mice was prepared for Western Blot analysis using an antibody against Arc (green fluorescence in Figure 5G) and an antibody against ß-actin as a loading control (red fluorescence). A band of fluorescence for endogenous Arc protein (N-Arc) is evident at around 50 kDa and a band of fluorescence for EGFP-Arc is evident at approximately 90 kDa in the transgenic mice, consistent with the predicted MW of the EGFP-Arc fusion protein (EGFP 32.7 kDa + Arc 55 kDa = 87.7 kDa). Quantification of the levels of fluorescence in the bands revealed that average levels of endogenous Arc were comparable in WT vs. *EGFP-Arc* +/+ mice, whereas levels of fluorescence for EGFP-Arc were about 7.6-fold higher than endogenous Arc. Thus, total Arc (endogenous + EGFP-Arc protein) is about 9-fold higher in the transgenic mice (two way ANOVA, *p* < 0.0001).

High basal levels of *EGFP-Arc* expression could reflect enhanced transcription, but this could occlude activity-dependent transcriptional activation; however, levels of *EGFP-Arc* mRNA were robustly elevated as a result of experience in the novel environment. The fact that transcription of *EGFP-Arc* mRNA in transgenic mice can be activated in a manner similar to endogenous *Arc* indicates that transcriptional activity is not saturated. Moreover, as we show below, EGFP-Arc protein is also not degraded as quickly as endogenous Arc, contributing to the net overexpression in the transgenic mice.

### Assessing activity- and protein synthesis-dependence of *Arc* mRNA degradation

We previously characterized activity- and protein synthesis-dependent *Arc* mRNA degradation in physiological experiments in rats (Farris et al., 2014). For this, we used the *ECS-Perforant Path stimulation* paradigm in which *Arc* is induced by an electroconvulsive seizure (ECS) and time is allowed for the mRNA to move throughout dendrites (1 h). In the absence of synaptic stimulation, *Arc* mRNA levels are high throughout the dendritic lamina (molecular layer) of the dentate gyrus. Following high frequency synaptic stimulation (HFS) of the perforant path, *Arc* mRNA localizes selectively in the activated dendritic lamina (Steward and Worley, 2001) and is depleted from non-activated portions of the dendrite due to activity-driven protein synthesis-dependent mRNA degradation (Farris et al., 2014).

Figures 6A–C illustrate these phenomena in WT mice using chromogen-based *in situ* hybridization. Following ECS, *Arc* mRNA is delivered throughout dendrites (Figure 6A). Perforant path stimulation then produced a distinct laminar pattern of labeling with higher levels of labeling in the zone of termination of the medial perforant path and lower levels of labeling in the outer molecular layer; arrows in Figure 6B indicate a distinct boundary between the two zones. Figure 6C illustrates a quantitative analysis of optical density (OD) across the granule cell layer and molecular layer with ECS only vs. ECS/PPStim.

A similar laminar pattern of labeling could be seen when WT mice were subjected to the ECS/PPStim paradigm and *Arc* mRNA levels were assessed by FISH. For example, Figure 6E illustrates the pattern of labeling for *Arc* mRNA in a WT mouse after ECS and 1 h of PPStim. However, the phenomenon was not seen as reliably in mice as in previous studies involving rats. For example, a distinct laminar pattern such as shown in Figure 6E was evident in 8/12 experiments in WT mice, whereas we observe striking lamination in every experiment in rats. This may be due to differences in the physiology of perforant path synapses in mice. For example, perforant path LTP itself is less reliable in mice than in rats (Steward et al., 2007). Also, although *Arc* mRNA transcription is strongly induced by ECS in WT mice, high frequency stimulation (HFS) of the perforant path alone typically does not strongly activate *Arc* transcription in large numbers of dentate granule cells (Steward et al., 2007).

In our previous study (Farris et al., 2014) the *ECS-Perforant Path stimulation* paradigm provided strong evidence for activity- and translation-dependent *Arc* mRNA degradation. If this degradation depends on splicing, it should not occur for *EGFP-Arc* mRNA. We tested this in a total of 7 *EGFP-Arc* mice that received ECS followed by HFS of the perforant path. When sections from *EGFP-Arc* mice were hybridized for *Arc* mRNA, *Arc* mRNA was present in a gradient in dendrites on the ECS only side (Figure 6G) and the laminar pattern of labeling was seen in some mice (Figure 6H), although quantitative assessments revealed that the effect was modest (Figure 6I). In contrast, hybridization with the probe for *EGFP* mRNA revealed intense labeling in cell bodies but very little labeling in the dendritic lamina on either the ECS only side (Figure 6J) or ECS-PPStim side (Figure 6K; and for quantification, see Figure 6L). Thus, the impairment of dendritic delivery of *EGFP-Arc* mRNA seen with ECS alone is also evident with ECS-PPStim. Because EGFP-Arc mRNA does not traffic out to dendrites in the time frame of the experiment, no inference can be drawn from the ECS-PPStim experiment in the Arc-EGFP +/+ mice about whether *EGFP-Arc* mRNA in dendrites is subject to activity-driven decay.

### Translation-dependence of *Arc* mRNA degradation

In our previous study in rats, we showed that activity-driven *Arc* mRNA degradation is abrogated in the presence of protein synthesis inhibitors, consistent with an NMD-like process (Farris et al., 2014). The first test was to locally-infuse cycloheximide (CHX) via a recording microelectrode during the stimulation phase of the ECS/PPStim paradigm. As predicted by the hypothesis that *Arc* mRNA degradation is via NMD, *Arc mRNA* levels were increased in the area of protein synthesis blockade. Although the largest percent increase was in the activated dendritic lamina, increases were also seen in the cell body lamina, so this experiment is not compromised by the fact that delivery of *EGFP-Arc mRNA* is impaired.

We used the same paradigm in mice and found similar results for endogenous *Arc* mRNA. Figures 7A,C illustrate *Arc mRNA* levels in the area of CHX infusion in a WT mouse; Figures 7B,D illustrate the contralateral side of the same section (ECS only); and Figures 7F,G illustrate *Arc* mRNA levels on the stimulated side in areas distant from the CHX infusion. The graphs in Figures 7E,H plot fluorescence intensity across the cell body and molecular layers. As previously documented in rats (Farris et al., 2014) *Arc* mRNA levels were elevated in the area of CHX blockade that extends for a diameter of about 1mm around the infusion site, especially in the activated dendritic lamina. Note that labeling is decreased in an area about 150 μm in diameter at the center of the infusion due to damage produced by the micropipette.

To test whether *EGFP-Arc* mRNA levels would also increase with CHX blockade, we carried out the same experiment as above in *EGFP-Arc* transgenic mice with one variation; rather than waiting 1 h after ECS before preparing mice for neurophysiology, we induced ECS and then immediately prepared mice for neurophysiology placing the CHX-filled micropipette as soon as possible. The time from ECS to placement of the CHX-filled micropipette was about 15min. Then we delivered HFS to the perforant path for 1 h. The rationale was to prevent translation of newly-transcribed *Arc* mRNA, which would prevent any translation dependent degradation. As illustrated in Figure 7I, hybridization with the probe for *Arc* mRNA revealed dendritic labeling with localization in the activated lamina (Figure 7I). However, hybridization of nearby sections with the probe for *GFP* mRNA revealed induction of expression due to ECS, but no labeling in the dendritic lamina (Figure 7J). It's important to reiterate that the probe for *Arc* mRNA recognizes both endogenous *Arc* and *EGFP-Arc* mRNAs. Because *EGFP-Arc* mRNA did not move into dendrites (Figure 7J) the labeling in the dendritic lamina with probes for *Arc* mRNA must reflect endogenous *Arc* mRNA.

The fact that dendritic delivery of *EGFP-Arc* mRNA is impaired even when translation of newly-transcribed mRNA is blocked (Figure 7J) is strong evidence against the possibility that dendritic delivery is impeded because of translation of the EGFP-Arc fusion protein *per se*. This points to the interpretation that deficient delivery is related to the fact that *EGFP-Arc* mRNA is not spliced and thus would not retain EJC proteins that otherwise might facilitate the shuttling of an *EGFP-Arc* mRNA complex out of the nucleus into the cytoplasm. A much less likely possibility that we cannot fully exclude is that dendritic delivery is impeded because of some sequestration signal in the *GFP* coding sequence that causes the mRNA to be largely retained in the cell body, thus over-riding the dendritic transport signal(s) that would otherwise mediate dendritic delivery of endogenous *Arc* mRNA.

### Inhibition of protein synthesis in awake-behaving mice does not lead to increases in *Arc* mRNA levels

The second way we tested protein synthesis-dependence of *Arc* mRNA in Farris et al. (2014) was to block protein synthesis during a learning experience (1 h exploration of a novel toy-filled environment). As predicted by translation-dependent decay, *Arc* mRNA levels were higher in rats treated with CHX in both the home cage and EE condition. Increases were qualitatively evident over both cell body and dendritic laminae of the hippocampus.

Here, we used the same basic approach in WT and *EGFP-Arc* transgenic mice. Mice were gently removed from their home cage and received injections of either CHX or saline, and were then either immediately returned to their home cage for 1 h, or were allowed to explore a novel environment for 1 h.

To our surprise, and in striking contrast to what we documented in rats, CHX did not lead to increased *Arc* mRNA levels in WT mice in either the home cage or after exploration of the novel environment (EE in figure panels). *Arc* mRNA levels are not noticeably higher in the mouse that received CHX (Compare Figure 8A, control, with Figure 8B, CHX); this is confirmed by quantitative analysis of fluorescence intensity over the CA1 pyramidal layer (Figure 8M; values for saline-treated HC and EE mice are the same as shown in Figure 1L. Values for individuals are shown along with bar graphs to indicate numbers of animals per condition). Although *Arc* mRNA levels were induced in WT mice that explored the novel environment [two-way ANOVA for home cage vs. EE: *F*_(1, 10)_ = 31.76, *p* = 0.0002], there were no differences between mice that received saline vs. CHX [two-way ANOVA: *F*_(1, 10)_ = 0.064, *p* = 0.805]. *Arc* mRNA levels were also comparable with and without CHX in the home cage (HC) group (Figure 8M). Similar results were seen in homozygous *EGFP-Arc* transgenic mice [Figure 8N, two-way ANOVA for home cage vs. EE: *F*_(1, 14)_ = 166.3, *p* < 0.0001], with no differences between saline vs. CHX groups [two-way ANOVA: *F*_(1, 14)_ = 1.333, *p* = 0.2676].

In our previous study (Farris et al., 2014), differences in *Arc* mRNA levels were qualitatively evident over both cell body and dendritic laminae but the quantitative analyses assessed the number of *Arc* mRNA puncta in the dendritic layer. Accordingly, we wondered whether we were missing an effect by measuring fluorescence intensity over the cell body lamina. Thus, we analyzed the number of *Arc* mRNA puncta per unit area of *stratum radiatum* in WT-EE mice using confocal stacks as in Figure 3. Again, there were the expected differences in *Arc* mRNA puncta between HC and EE groups, but no differences with and without CHX. Puncta counts were: WT-HC saline-535 ± 91, WT-HC CHX-587 ± 31, WT-EE saline-1559 ± 515, WT-EE CHX-1490 ± 693.

Given the absence of an effect of CHX in WT mice, it is not surprising that blockade of protein synthesis also did not lead to increases in *EGFP-Arc* mRNA levels in homozygous *EGFP-Arc* transgenic mice that were allowed to explore the enriched environment. Figures 8C,D illustrate levels of *EGFP-Arc* mRNA following hybridization with probes for GFP (see Figure 8N for quantification). *EGFP-Arc* mRNA levels were somewhat elevated in homozygous *EGFP-Arc* transgenic mice that received CHX and were returned to their home cage (Figure 8N), but differences were not statistically significant (*t* = 2.71, *df* = 6, *p* = 0.412).

### Persistence of EGFP-Arc protein following CHX-treatment

In WT mice, blockade of protein synthesis with CHX prevents the increases in Arc protein that are otherwise seen with exploration of an enriched environment and also leads to loss of immunostaining, so that the Arc-positive dentate granule cells that are normally present are no longer evident (Compare Figure 8E, saline with Figure 8F, CHX-treated). This run-down of Arc protein is a convenient way to confirm the effectiveness of CHX treatment. Surprisingly, EGFP-Arc protein did not run down with CHX treatment in the same way as endogenous Arc. In *EGFP-Arc* transgenic mice, although CHX treatment did block the increases in immunostaining that otherwise occur after exploration of a novel environment, this did not lead to the complete disappearance of Arc-positive neurons as in wildtype mice (compare Figure 8G with Figure 8H and Figure 8I with Figure 8J). It was noteworthy in the high magnification views in Figures 8I,J that CHX did block the increases in Arc protein in the dendrites of dentate granule cells. This suggests that the appearance of EGFP-Arc protein in dendrites occurs with kinetics similar to that of endogenous Arc protein in the wildtype mice. The relative absence of *EGFP-Arc* mRNA means that this occurs via a mechanism other than local protein synthesis.

To exclude the possibility that protein degradation machineries were overwhelmed by induced overexpression of EGFP-Arc proteins, we tested whether CHX caused a rundown of other IEG proteins in *EGFP-Arc* transgenic mice. For this, sections adjacent to those taken from the mice shown in Figures 8G,H were immunostained for c-Fos (Figures 8K,L). There were the expected number of c-Fos positive granule cells in the mouse that received saline before exploration of the novel environment (Figure 8K; higher power views of dentate granule cells are shown in Figure 8O), and only a few c-Fos labeled granule cells were present in the mouse that received CHX (Figures 8L,P). Thus, degradation of endogenous Arc and c-Fos proteins was intact and unaffected 1 h following CHX-treatment whereas EGFP-Arc protein persisted, at least within the cell bodies of the dentate gyrus granule cells. The apparent persistence of EGFP-Arc protein may reflect a quantitative difference (near 8-fold overexpression), a qualitative difference (delayed turnover due to slower local decay kinetics), or both. One possible explanation for the latter is that the presence of EGFP in the EGFP-Arc recombinant protein alters degradation kinetics.

## Discussion

The original goal of this study was to test the hypothesis that activity- and translation-dependent degradation of *Arc* mRNA occurs via NMD, which depends on the presence of EJC protein binding in the 3′UTR. The approach used transgenic mice carrying an *EGFP-Arc* transgene constructed from *Arc* cDNA, which lacks two introns in the 3′UTR that are present in the endogenous *Arc* gene. The canonical signal for NMD is an EJC downstream of a stop codon, so *EGFP-Arc* mRNA should not be susceptible to NMD. Our findings were consistent with the NMD hypothesis: (1) *EGFP-Arc* mRNA decayed more slowly following transcriptional blockade than endogenous *Arc* mRNA indicating impaired mRNA degradation; (2) Local infusion of CHX in the ECS/PPStim paradigm led to increases in endogenous *Arc* mRNA in the area of protein synthesis blockade, consistent with translation-dependent *Arc* mRNA degradation.

Our results also revealed an unexpected and striking impairment of dendritic delivery of *EGFP-Arc* mRNA. This suggests the intriguing possibility that the presence of an intact splice junction and accompanying EJC proteins might provide an additional activity-regulated mechanism to enhance delivery of *Arc* mRNA into dendrites. In what follows, we discuss the caveats of the experiments and un-expected findings that provide new insights into *Arc* mRNA dynamics.

### Role of nonsense mediated decay (NMD) in *Arc* mRNA turnover

Previous studies have documented that *Arc* mRNA is a canonical candidate for NMD because of the presence of splice junction sites in the 3′UTR downstream of the stop codon (Giorgi et al., 2007). Proteins of the exon junction complex (EJC) remain bound to *Arc* mRNA as it moves into the cytoplasm and EJC's downstream of a stop codon are the canonical signal for triggering NMD when a translating ribosome reaches the stop codon. In support of the NMD hypothesis, our previous studies in rats (Farris et al., 2014) showed that *Arc* mRNA degradation is attenuated by protein synthesis inhibitors and accelerated by synaptic stimulation.

The *EGFP-Arc* transgene was created by inserting a monomeric *EGFP* cDNA into mouse *Arc* cDNA with its 5′ and 3′ UTRs under the control of the *Arc*7000 promoter (pGL4.11-*Arc*7000-m*EGFP-Arc*-UTRs). *Arc* cDNA lacks the introns in the 3′UTR so the transcript never undergoes splicing. If *Arc* mRNA turnover is via an NMD-like mechanism, then: (1) *EGFP-Arc* mRNA should be degraded more slowly than endogenous *Arc* mRNA; (2) Inhibition of protein synthesis should not lead to increases in *EGFP-Arc* mRNA levels seen with endogenous *Arc* mRNA (Farris et al., 2014). Our results confirmed the first prediction; assessment of rundown of induced *Arc* mRNA levels after blockade with the transcription inhibitor actinomycin-D revealed that *EGFP-Arc* mRNA is degraded more slowly than endogenous *Arc* mRNA.

The test of the second prediction was compromised for two reasons. First, local infusion of CHX in the ECS/PPStim paradigm did lead to increases in endogenous *Arc* mRNA in the activated dendritic lamina; however, *EGFP-Arc* mRNA did not enter dendrites even in the presence of CHX, so it was not possible to asses for increases in dendrites due to protein synthesis inhibition. Second, inhibition of protein synthesis with CHX in awake behaving mice did not lead to increases in endogenous *Arc* mRNA, so this test of translation-dependence was not relevant in mice. It is unknown why Arc mRNA turnover in mice is less translation-dependent than is the case in rats.

Although the tests of the hypothesis yielded results that were more complicated than expected, the evidence does support the hypothesis that *Arc* mRNA decay is either regulated by splicing of the 3′UTR (perhaps by EJC proteins that accompany *Arc* mRNA into the cytoplasm) or that insertion of the coding sequence for EGFP disrupts some signal in the coding region that regulates mRNA decay. Thus, a mechanism with NMD-like characteristics contributes to *Arc* mRNA degradation, and this mechanism is disrupted in *EGFP-Arc* transgenic mice, disrupting normal IEG dynamics.

Although our results indicate that the presence of introns plays a role in regulating mRNA decay, *EGFP-Arc* mRNA is degraded, albeit with a slower time course, indicating that mechanisms other than NMD also contribute. In this regard, it is noteworthy that a recent study reports that the presence of the 3′UTR sequence called the “dendritic targeting element (DTE)” in fusion transcripts also confers destabilizing activity independent of NMD (Ninomiya et al., 2016).

### Impaired dendritic delivery of newly-synthesized *EGFP-Arc* mRNA

An unexpected discovery was the striking difference in the delivery of endogenous *Arc* mRNA vs. *EGFP-Arc* mRNA into dendrites even after massive induction by ECS. This suggests a deficiency in steps leading to dendritic transport of newly-synthesized *EGFP-Arc* mRNA. Dendritic transport of many mRNAs is thought to depend on sequences in the 3′UTR, but after splicing of endogenous *Arc* mRNA, the sequence of the UTR would be identical to that of *EGFP-Arc* mRNA; thus, 3′UTR sequence alone is not sufficient to mediate efficient dendritic transport of *EGFP-Arc* mRNA. Possible explanations include: (1) Rapid dendritic delivery after induction may depend on one or more EJC proteins that are retained with endogenous *Arc* mRNA after 3′UTR splicing; (2) Efficient dendritic delivery may be disrupted by the presence of the sequence for EGFP in the coding region.

The fact that *EGFP-Arc* mRNA is not delivered into dendrites even when translation of newly-transcribed *EGFP-Arc* mRNA is blocked (Figure 7J) argues against the possibility that dendritic delivery is impeded because of translation of the EGFP-Arc fusion protein. It is also unlikely that dendritic delivery is impeded because the EGFP coding sequence contains some signal that causes the mRNA to be retained in the cell body because EGFP was previously used with success as a reporter for characterizing the dendritic targeting sequence in the fully spliced 3′UTR of *Arc* mRNA (Kobayashi et al., 2005). Thus, the most likely interpretation is that impaired delivery is related to the fact that *EGFP-Arc* mRNA is not spliced and thus would not retain EJC proteins. It is noteworthy that localization of *oskar* mRNA in the posterior pole of the Drosophila oocyte also depends on splicing and presumably deposition of EJC proteins upstream of the exon-exon junction (Hachet and Ephrussi, 2004). The authors propose that splicing is important for regulating ribonucleoprotein complex assembly and organization for cytoplasmic localization. In this regard, the fact that *EGFP-Arc* mRNA is present in globs in the perinuclear cytoplasm rather than discrete puncta is consistent with the idea that splicing plays a role in packaging the RNA into granules or in granule trafficking thereafter. It may be that splicing and deposition of EJC proteins is a more general mechanism that is critical for mRNA localization in both developing systems and mature neurons.

Our previous studies using live cell imaging document that fusion transcripts with the *Arc* 3′UTR and MS2 binding sites are transported in dendrites (Dynes and Steward, 2008) and localize with a high degree of precision at the base of dendritic spines (Dynes and Steward, 2012). The DNA constructs for these fusion transcripts were also made from *Arc* cDNA, and so would not undergo splicing. Thus, splicing and/or the presence of EJC proteins at the splice site are not absolutely required for dendritic transport of transcripts containing the *Arc* 3′UTR or selective localization at spines.

### Selective localization of newly-synthesized *Arc* mRNA at active synapses

*Arc* mRNA localizes selectively in dendritic domains contacted by active synapses (Steward et al., 1998), due mainly to selective accumulation of newly-synthesized *Arc* mRNA (Farris et al., 2014). However, there was no hint of accumulation of newly-synthesized *EGFP-Arc* mRNA in the activated dendritic lamina in any of the transgenic mice tested. Thus, signals induced by strong synaptic activation that enhance dendritic delivery of endogenous *Arc* mRNA are not sufficient to overcome the impaired dendritic transport of newly-synthesized *EGFP-Arc* mRNA.

### Levels of EGFP-Arc protein are high throughout dendrites despite low levels of *EGFP-Arc* mRNA

It is often assumed that high levels of Arc protein in dendrites depend on the presence and local translation of *Arc* mRNA. Despite impaired dendritic delivery of *EGFP-Arc* mRNA, however, EGFP-Arc protein levels were very high in the dendrites of some neuron types under resting conditions, particularly neurons in the CA1 region of the hippocampus (Figure 5E). EGFP-Arc protein does not run down as quickly after protein synthesis inhibition as endogenous Arc, so high levels of expression of EGFP/Arc protein may be due to a combination of delayed degradation of *EGFP-Arc* mRNA and delayed degradation of EGFP-Arc protein.

It is noteworthy that increases in EGFP-Arc protein levels with ECS- or repetitive PP stimulation were mainly in the cell body and proximal dendritic regions (for example, Figure 5F). These results suggest two independent, yet not mutually exclusive possibilities: (1) In hippocampal neurons that express EGFP-Arc at high levels under resting conditions, EGFP-Arc protein can reach the distal tips of dendrites even with only a small contribution of local translation of dendritically delivered *EGFP-Arc* mRNA. (2) However, activity-dependent increases with seizures, synaptic stimulation or learning occur mainly in subcellular domains in which *EGFP-Arc* mRNA is localized. This may be because such activity regimes favor local protein translation.

### Implications for previous studies involving *EGFP-Arc* transgenic mice

The *EGFP-Arc* transgenic mice used here express an EGFP-Arc fusion protein, and thus differ from other Arc-GFP reporter transgenic mouse lines. In one other line, the coding region of *Arc* is entirely replaced with a *GFP* gene, thus causing an *Arc* knockout (Wang et al., 2006). In another series of mouse lines, destabilized GFP was driven by an *Arc* promoter in a bacterial artificial chromosome (BAC) (Grinevich et al., 2009) or as a transgene (Eguchi and Yamaguchi, 2009; Vousden et al., 2015), with variable amounts of activity-dependent inducibility and background expression. These lines have subsequently been used to identify neurons in which *Arc* expression has been induced, e.g., Jakkamsetti et al. (2013) but the reporter is not an Arc-GFP fusion protein and thus would not be germane to elucidating the role of different sequences in affecting dendritic transport. In contrast, the *Arc* transgenic line used here has both copies of the endogenous *Arc* gene as well as multiple copies of the *EGFP-Arc* transgene, and over-expresses total *Arc* mRNA and protein (endogenous Arc + EGFP-Arc). Accordingly, the *EGFP-Arc* tg/tg transgenic mice will be useful models for studies of the physiological and behavioral consequences of Arc over-expression coupled with alterations in dendritic transport. In this regard, a recent study has reported that one type of ocular dominance plasticity persists in adult mice from an mCherry-Arc Tg line with approximately 8-fold overexpression of Arc in comparison to WT (Jenks et al., 2017).

## Ethics statement

Ethics Statement Involving Animal Subjects. This study was carried out in accordance with the recommendations of IACUC Protocol Number 1999-2092: Mechanisms Underlying mRNA Transport to Synapses. The protocol was approved by The University of California, Irvine Office of Research, Institutional Animal Care and Use Committee (IACUC). The University of California, Irvine has an approved Animal Welfare Assurance (#A3416.01) on file with the NIH Office of Laboratory Animal Welfare. Part of the live animal experiments were performed at Kyoto University and at the University of Tokyo, with permits from the Institutional Animal Care and Use Committees of Kyoto University and of the University of Tokyo.

## Author contributions

OS: Responsible for overall design of the study, carried out experiments, wrote the manuscript with input from all authors. KMY: Carried out *in situ* hybridization, immunohistochemistry, Western blot, analyzed data. SF: Developed techniques, contributed to writing of the manuscript. PSP: Confocal microscopy, quantitative analyses of images. PW: Contributed to the design of the study. KO: Determined chromosomal location of transgene and copy number. HO: Created transgenic mice, provided data, contributed to writing of the manuscript. HB: Created transgenic mice, provided data, contributed to writing of the manuscript.

### Conflict of interest statement

The authors declare that the research was conducted in the absence of any commercial or financial relationships that could be construed as a potential conflict of interest.
